# The tectal melanocortin system modulates energy-dependent visual avoidance behavior in zebrafish

**DOI:** 10.1016/j.isci.2026.116095

**Published:** 2026-05-22

**Authors:** Madhuri Puvvada, Tim Hladnik, Yue Zhang, Fabian Svara, Silke Lemmens, Louise von Gersdorff Jørgensen, Kevin Briggman, Aristides Arrenberg, Dominique Förster, Matthias Hammerschmidt

**Affiliations:** 1 Institute of Zoology, University of Cologne, Cologne, Germany; 2 Werner Reichardt Centre for Integrative Neuroscience, Tuebingen, Germany; 3 Max Planck Institute for Neurobiology of Behavior – Caesar, Bonn, Germany; 4 Department of Veterinary and Animal Sciences, Faculty of Health and Medical Sciences, University of Copenhagen, Frederiksberg, Denmark; 5 Department of Neurology, Faculty of Medicine and University Hospital Cologne, University of Cologne, Cologne, Germany; 6 Center for Molecular Medicine Cologne (CMMC), Cologne, Germany

**Keywords:** biological sciences

## Abstract

Energy homeostasis depends on both food intake and behavioral control of energy expenditure. The hypothalamic melanocortin system is classically associated with feeding regulation, but its broader behavioral roles remain less defined. Using larval zebrafish, we show that hypothalamic pro-opiomelanocortin (pomca)-expressing neurons project to the tectum and target neurons expressing the melanocortin receptor Mc4r. Rather than altering prey consumption, melanocortin signaling in this circuit modulates visually guided avoidance of small stimuli, an energy-demanding behavior. Sated larvae, with higher energy reserves, avoid small objects (prey or other, potentially harmful, protozoa) more readily, while hungry larvae do not. Importantly, disrupting Mc4r signaling in sated fish reduces this avoidance behavior, indicating that the melanocortin system gates energy expenditure decisions based on internal state. These findings uncover a previously undescribed function for hypothalamic melanocortin signaling in tuning sensorimotor responses to visual stimuli, not to regulate feeding per se but to modulate behavioral energy allocation.

## Highlights

• Pomc neurons project to the tectum targeting Mc4r+ melanocortin neurons
• Melanocortin signaling shifts tectal size tuning toward larger stimuli
• Mc4r signaling promotes avoidance of small visual objects in sated larvae
• Tectal melanocortin signaling biases behavior without altering food intake

## Graphical abstract

**Figure:**
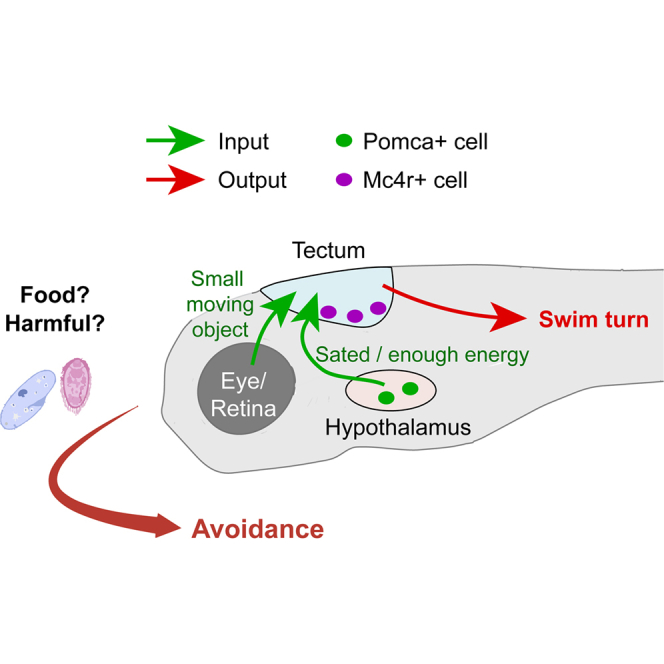

## Introduction

Foraging is a fundamental behavior that requires animals to continuously evaluate sensory information in light of their internal physiological state. Rather than simply maximizing food intake, animals must balance potential energetic gains against costs such as energy expenditure, predation risk, and exposure to harmful environmental factors.^1^ This balance is dynamically shaped by internal energy reserves: hungry animals tend to adopt risk-prone, energy-conserving strategies that favor food acquisition, whereas sated animals are more likely to engage in energetically costly avoidance behaviors, reflecting a shift in the perceived salience and valence of sensory stimuli.^2^^,^^3^ Such state-dependent modulation of behavior has been observed across species and is particularly evident in visually guided foraging, where identical stimuli can elicit approach or avoidance depending on the animal’s metabolic condition.

The neural mechanisms that couple internal energy state to sensory processing and behavioral decision-making remain incompletely understood. A central candidate for mediating this interaction is the hypothalamic melanocortin system, a highly conserved regulator of energy homeostasis in vertebrates. This system is classically defined by the antagonistic activity of pro-opiomelanocortin (Pomc)-expressing neurons and agouti-related peptide (Agrp)-expressing neurons in the hypothalamus. Pomc neurons, activated under conditions of energy surplus, release α-melanocyte-stimulating hormone (αMSH), which acts on melanocortin receptors such as melanocortin 4 receptor (Mc4r) to suppress food intake and promote energy expenditure, whereas Agrp neurons are activated during energy deficit and drive feeding while reducing energy expenditure.^4^^,^^5^^,^^6^^,^^7^^,^^8^ While extensive work has established the role of melanocortin signaling in hypothalamic and hindbrain circuits controlling feeding and metabolism, its potential influence on higher order sensory processing and sensorimotor transformations has remained largely unexplored.

Emerging evidence suggests that internal state signals can directly modulate sensory processing, including visual circuits, by altering neuronal gain and selectivity.^3^^,^^9^ In zebrafish larvae, the optic tectum, which is homologous to the mammalian superior colliculus, integrates visual inputs and transforms them into motor outputs such as approach and avoidance. Tectal circuits encode object features like size and motion, enabling discrimination between prey and predators. Notably, these visuomotor responses are strongly modulated by feeding state,^3^ suggesting that internal energy signals may directly influence tectal processing. However, it remains unclear whether metabolic signals such as melanocortin signaling directly access and modulate visual centers like the tectum or act solely through canonical feeding circuits.

Here, we address this question using larval zebrafish by identifying a projection from hypothalamic Pomc neurons to the optic tectum and showing that tectal neurons express the melanocortin receptor Mc4r. Functionally, melanocortin signaling in the tectum does not regulate prey consumption but biases behavioral decisions by promoting avoidance of small visual stimuli in energy-replete states. Together, our findings reveal a previously unrecognized role of hypothalamic melanocortin signaling in tuning sensorimotor processing and energy-dependent action selection.

## Results

### Hypothalamic Pomca neurons project to the zebrafish tectum

**Figure 1:**
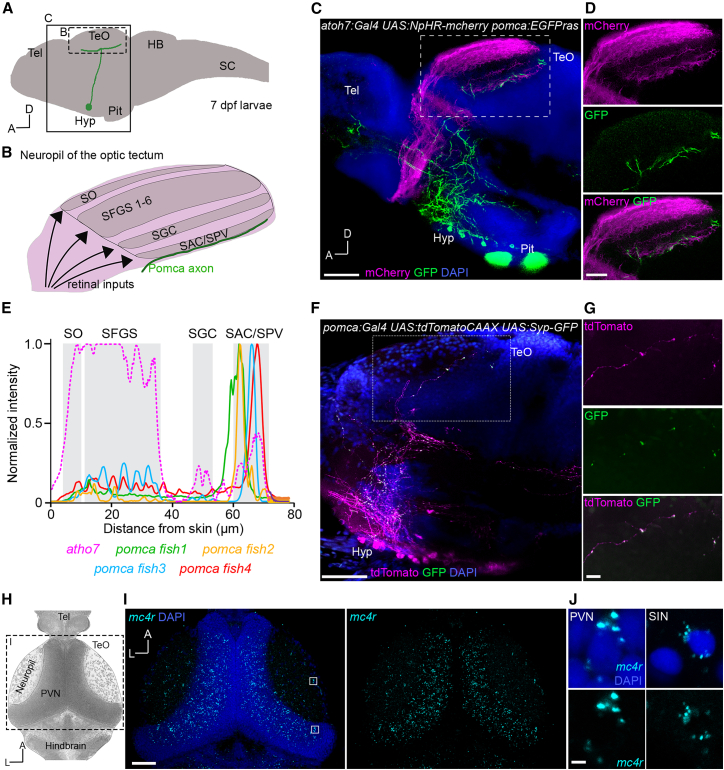
Hypothalamic Pomca neurons project axons to the tectum in zebrafish larvae (A) Schematic of a zebrafish larval brain at 7 dpf (side view). Imaged regions are outlined. HB, hindbrain; Hyp, hypothalamus; Pit, pituitary; SC, spinal cord; Tel, telencephalon; TeO, optic tectum. (B) Laminar organization of the tectal neuropil with Pomca axons illustrated in green. SAC/SPV, stratum album centrale/stratum periventriculare; SFGS, stratum fibrosum et griseum superficiale; SGC, stratum griseum centrale; SO, stratum opticum. (C) Single plane side view of an *atoh7:Gal4 UAS:NpHR-mcherry pomca:EGFPras* transgenic larva at 7 dpf, stained against GFP and RFP. Scale bars, 50 μm, *n* = 9. (D) Close-up of boxed region in (C), showing Pomca axons in the tectal neuropil. Scale bars, 20 μm. (E) Fluorescence intensity plots of four 7 dpf larvae expressing *pomca:EGFPras*, co-registered using *atoh7:Gal4 UAS:NpHR-mCherry* expression, plotted as a function of distance from the skin. (F) Immunofluorescence for GFP and tdTomato on a *pomca:Gal4 UAS:tdTomatoCAAX UAS:Syp-GFP* transgenic larva at 7 dpf. Scale bars, 50 μm. *n* = 4 fish. (G) Expanded view of highlighted region in (F). Scale bars, 10 μm. (H) Illustration of dorsal view on midbrain area in a 7 dpf larva. Imaged region for (I) is outlined. (I) Maximum intensity projection (stack size = 5 μm) representing expression of *mc4r* in the tectum of a 7 dpf larva. *n* = 12 fish. Scale bars, 50 μm. (J) Single plane close-up of boxed regions in (I), showing *mc4r* expression in tectal PVNs and superficial interneurons (SINs). Scale bars, 2 μm. DAPI counterstaining is shown in (C), (F), (I), and (J). See also Figures S1 and S2.

*pomc*-expressing and antagonistic agrp-expressing neurons in the hypothalamus are central parts of the melanocortin circuitry, which are activated (*pomc*) or inhibited (*agrp*) by energy surfeit and which in turn inhibit (*pomc*) or promote (*agrp*) food intake, while promoting (*pomc*) or inhibiting (*agrp*) energy expenditure.^4^ In order to dissect axonal targets of satiety-mediating Pomc neurons possibly involved in modulating sensory processing, we inspected a previously published transgenic zebrafish line, in which a membrane-tagged GFP is under direct control of the *pomca* promoter.^10^ Besides various other target areas in the larval brain, we identified Pomca cells located in the nucleus lateralis tuberis (NLT) of the rostral hypothalamus, which showed direct axonal projections to the tectum, the largest visual processing area in fish, homologous to the superior colliculus in mammals^11^ (Figures 1A–1C). Co-labeling with retinal ganglion cells (RGCs) revealed that these Pomca axons entered the tectum ventrally from the anterior and arborized and terminated in the deepest tectal layer, the stratum album centrale/stratum periventriculare (SAC/SPV; Figures 1D and 1E). Axons were spanning the entire anterior-posterior axis of this layer. Importantly, the presence of Pomca axonal innervation in the tectum was consistently observed in every imaged fish at 7 days post-fertilization (dpf), indicating that this projection is a robust and stereotyped feature of the larval Pomca circuitry. These findings could also be reproduced using an independent transgenic line, in which the Gal4 transcriptional activator is expressed in Pomca cells^10^ (Figure 1F). Furthermore, we could confirm that Pomca axons form presynaptic specializations in the tectum, labeled by Syp-GFP (Figures 1F and 1G). Syp-GFP punctae were found along the entire axonal arborization in SAC/SPV. In addition, we found Pomca axons passing and synapsing in extratectal retinal arborization fields (AFs), specifically in AF4 and AF9 (Figure S1). Interestingly, we did not observe innervations or presynaptic punctae in the neuropil of, or near, the parvocellular superficial pretectal nucleus (PSp, AF7), which has functionally been implicated in prey recognition and hunting behavior^12^^,^^13^ (Figure S1).

### The melanocortin receptor Mc4r is expressed in tectal cells

Pomc neurons release the neuropeptide αMSH, which acts anorexigenic on downstream cells that express *mc4r*.^4^ To reveal direct cellular targets of αMSH-releasing Pomca axons in the tectum, we utilized RNA *in situ* hybridization chain reaction (HCR)^14^ to identify *mc4r* expression in 7 dpf larval zebrafish. We consistently observed *mc4r* expression in tectal periventricular neurons (PVNs), evenly distributed within the tectum, as well as sparse expression in the tectal neuropil originating from superficial and neuropil interneurons (SINs and NINs) (Figures 1H–1J). To identify the neurotransmitter identity of *mc4r*-expressing tectal cells, we performed multiplexed HCR using markers for excitatory and inhibitory neurons, known to be present in the tectum: vesicular glutamate transporter 2a (*vglut2a*), and glutamate decarboxylase 1b (*gad1b*), respectively. Interestingly, we found that both glutamatergic and GABAergic cell types were co-expressing *mc4r* in the tectum (Figure S2).

### The Mc4r agonist MTII increases avoidance of prey-like stimuli

Previous studies in zebrafish could show that the behavioral responses to appetitive versus aversive visual stimuli could be modulated by the feeding state of the animal.^3^^,^^9^ Our identification of αMSH-releasing axons and *mc4r*-expressing neurons in the tectum suggests that melanocortin signaling might be directly involved in this modulation of visual processing and behavioral decision-making.

**Figure 2:**
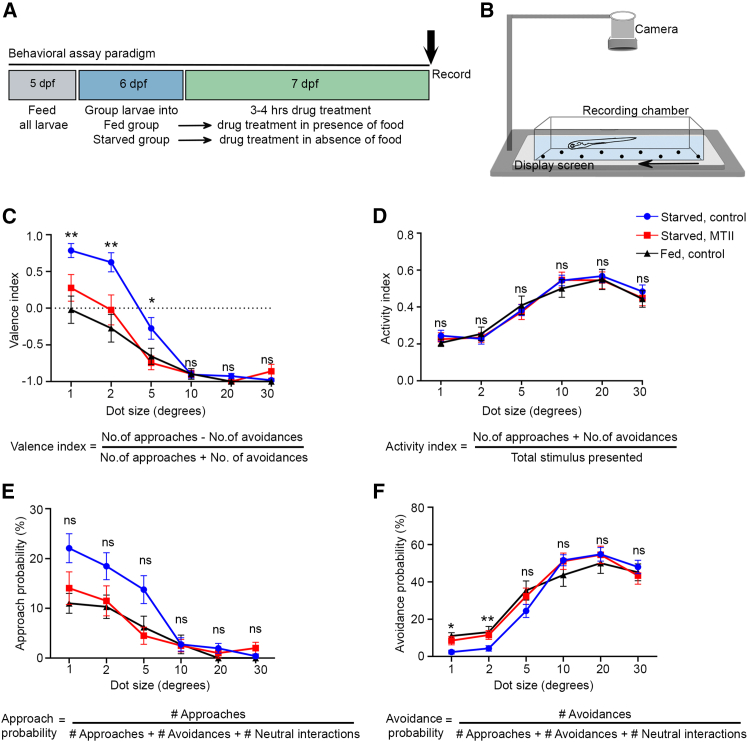
Pharmacological interference with the melanocortin system affects decision to approach or avoid visual prey-like stimuli (A) Feeding protocol and drug treatment paradigm used for size-discrimination behavioral assay. (B) Schematic of behavioral setup used for the size-discrimination assay. Recording chamber with a freely moving 7 dpf larva, interacting with moving visual stimuli (dots), displayed on the screen placed below. (C–F) Dependence of valence (C), activity (D), approach (E), and avoidance (F) on dot size in starved, fed, and starved MTII-treated larvae. Data presented as mean ± SEM. *n*_starved, control_ = 28, *n*_fed, control_ = 16, *n*_starved, MTII_ = 22. ∗*p* < 0.05, ∗∗*p* < 0.01, ∗∗∗*p* < 0.001; ns, not significant. *t* test with Benjamini-Hochberg correction. Asterisks indicate statistical significance for control-starved and MTII-starved comparisons. See Table 1 for all statistical comparisons. See also Figures S3–S5; Videos S1, S2, and S3.

To test this, we measured approach vs. avoidance behavior in 7 dpf larval zebrafish using an established size discrimination assay,^15^ while pharmacologically targeting the melanocortin system (Figures 2A and 2B). We employed automated behavioral analysis to distinguish avoidance swims from normal spontaneous swimming.^16^ This analysis revealed that avoidance of small objects is predominantly mediated by slower swims, whereas larger objects elicit faster swim responses, as reflected by differences in swim velocity (Figure S3; Videos S1, S2, and S3). In accordance with previous studies in zebrafish,^8^^,^^17^ we confirmed that our feeding paradigm influences the melanocortin system by enhanced expression of *pomca* in hypothalamic neurons of sated larvae and elevated expression of *agrp* in starved larvae (Figure S4). Consistent with an earlier report,^3^ we found that hungry larvae preferred small, prey-like stimuli and took more risk in approaching intermediate-sized stimuli by reducing avoidance when compared to sated larvae (Figure 2C). Strikingly, pharmacological treatment of hungry larvae with melanotan-II (MTII), an αMSH analog and Mc4r agonist, reduced the preference for small- and intermediate-sized visual stimuli (Figure 2C), without changing the general response probability to the presented stimuli (Figure 2D). This shift in behavioral choice upon MTII treatment mimics responses observed in sated larvae (Figure 2C). Of note, this treatment primarily changed the probability of the larvae to avoid visual stimuli of small sizes, while the approach probability was not altered significantly (Figures 2E, 2F, and S5; Table 1). Importantly, MTII treatment did not alter swim velocity or total distance traveled, indicating that the observed effects are specific to behavioral decision-making (Figure S3). These findings indicate a strong energy state-dependent modulation of behavioral choice to approach or avoid a visual cue, which is regulated by the melanocortin system.

**Table 1:** Statistical analysis of size-discrimination behavior in starved, fed, and MTII-treated larvae

Figure 2C	Valence index					
Groups compared	Circle size (°)					
Control starved vs. control fed	1	2	5	10	20	30
	0.0001	0.0002	0.081	0.908	0.130	0.455
Control starved vs. MTII starved	0.010	0.009	0.015	0.889	0.076	0.166
Control fed vs. MTII starved	0.264	0.402	0.542	0.990	–	0.221

Figure 2D	Activity index					
Groups compared	Circle size (°)					
Control starved vs. control fed	1	2	5	10	20	30
	0.366	0.579	0.629	0.414	0.775	0.495
Control starved vs. MTII starved	0.682	0.858	0.851	0.954	0.689	0.530
Control fed vs. MTII starved	0.660	0.658	0.580	0.478	0.945	0.933

Figure 2E	Approach probability					
Groups compared	Circle size (°)					
Control starved vs. control fed	1	2	5	10	20	30
	0.011	0.049	0.072	0.999	0.143	0.456
Control starved vs. MTII starved	0.074	0.094	0.012	0.894	0.502	0.161
Control fed vs. MTII starved	0.475	0.773	0.547	0.909	0.401	0.157

Figure 2F	Avoidance probability					
Groups compared	Circle size (°)					
Control starved vs. control fed	1	2	5	10	20	30
	0.00004	0.005	0.079	0.196	0.464	0.628
Control starved vs. MTII starved	0.011	0.009	0.167	0.921	0.961	0.425
Control fed vs. MTII starved	0.420	0.685	0.650	0.319	0.542	0.792

Figure S5A	Neutral interaction probability					
Groups compared	Circle size (°)					
Control starved vs. control fed	1	2	5	10	20	30
	0.554	0.898	0.534	0.191	0.296	0.579
Control starved vs. MTII starved	0.461	0.876	0.529	0.676	0.693	0.513
Control fed vs. MTII starved	0.849	0.999	0.312	0.402	0.510	0.968

Video S1. Approach behavior of a 7 dpf zebrafish larva toward a 1° visual stimulus, related to Figure 2 Playback frame rate is reduced by 50%.

Video S2. Avoidance behavior of a 7 dpf zebrafish larva toward a 1° visual stimulus, related to Figure 2 Playback frame rate is reduced by 50%.

Video S3. Avoidance behavior of a 7 dpf zebrafish larva toward a 20° visual stimulus, related to Figure 2 Playback frame rate is reduced by 50%.

### Activation of melanocortin signaling shifts tectal size tuning to larger visual stimuli

**Figure 3:**
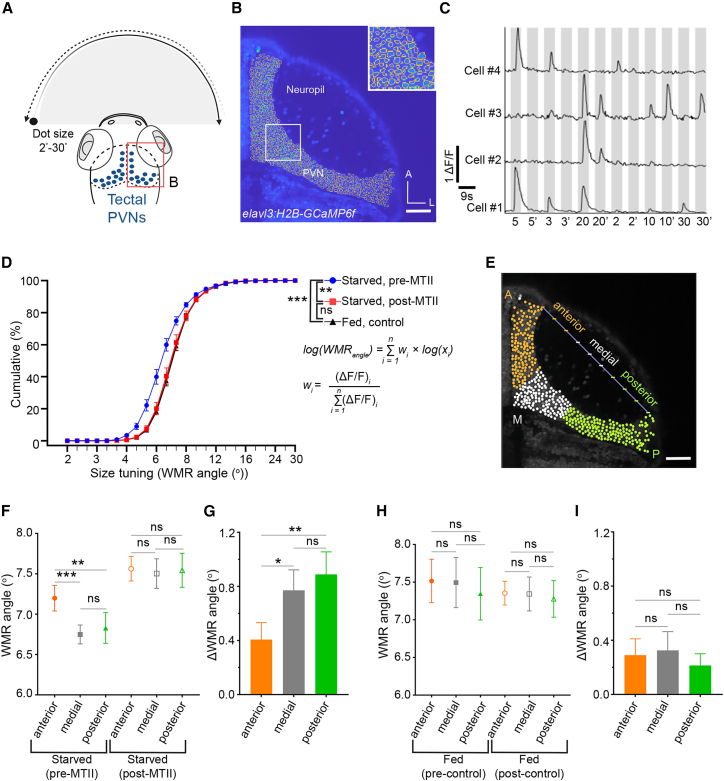
The melanocortin system modulates size tuning of tectal neurons along the anterior-posterior axis (A) Schematic showing presentation of visual stimuli on the frontal visual field of zebrafish larvae embedded in agarose, in both clockwise and anti-clockwise directions, and simultaneous recording of calcium activity in tectal neurons. Imaged region outlined in red. (B) Image showing tectal neurons (PVNs) selected for analysis of calcium imaging in *elavl3*:*H2B*-*GCaMP6f* at 7 dpf, with close-up of boxed region in the upper right. Scale bars, 50 μm. (C) Normalized ΔF/F traces for exemplary tectal neurons responding to visual stimuli. Gray bars indicate stimulus ON frames (9 s), and the gaps in between indicate stimulus OFF frames (5 s), with object size in degrees, and clockwise and anti-clockwise (‘) directions indicated below. (D) Cumulative percentages of weighted mean response (WMR) angles of tectal neurons in *elavl3:H2B-GCaMP6f* larvae before and after MTII treatment in starved (*n* = 7 fish) and in control-fed (*n* = 5 fish) conditions. Control larvae were mounted in agarose for the same duration of drug treatment (see STAR Methods). (E) Grouping of tectal neurons along the anterior-posterior (A-P) axis. Scale bars, 50 μm. (F and G) WMR angles (F) and difference in WMR angles (G) of tectal neurons before and after MTII treatment in starved larvae segregated along the A-P axis (*n* = 7 fish). (H and I) Same graphs as in (F) and (G) but for untreated fed (control) larvae (*n* = 3 fish). ∗*p* < 0.05, ∗∗*p* < 0.01, ∗∗∗*p* < 0.001; ns, not significant. Paired Wilcoxon signed-rank test was performed on median WMR angle (defined as the WMR angle at which 50% neurons were responsive), derived from the cumulative WMR distributions, or Mann-Whitney’s test was used for independent groups (D). A two-way mixed-effects model of ANOVA, followed by Tukey’s multiple-comparisons test (F and H), paired Wilcoxon signed-rank test, and Mann-Whitney’s test, was used for independent groups (G and I). Data are presented as mean ± SEM. See also Figures S6–S8.

The decision between avoidance and approach behaviors in zebrafish is established in the tectum.^15^ Thus, we next investigated whether the observed switch in behavioral preference upon activating the melanocortin system could result from modulated response properties of tectal neurons. To test this hypothesis, we used a full-surround visual stimulation setup^18^^,^^19^ (Figure S6) to record the activity of tectal neurons with single-cell resolution, in larvae expressing the nuclear-localized calcium sensor GCaMP6f (Figures 3A–3C). In alignment with an earlier study,^3^ we found that satiety shifted the overall population response of tectal neurons to larger visual stimuli compared to hungry larvae (Figure 3D). In line with our hypothesis, pharmacological treatment of starved larvae with the Mc4r agonist MTII also shifted the size tuning of tectal cells toward larger visual stimuli, similar to the shift observed in sated larvae (Figure 3D). Mock-treated control larvae did not display such a change in population response (Figure S7). Analysis of SINs and NINs revealed no significant MTII-dependent changes in their size tuning (Figure S8). These results suggest that melanocortin-dependent modulation of visuomotor transformation in tectal PVNs is responsible for the previously observed changes in behavior.

### The melanocortin system alters size tuning differentially along the tectal anterior-posterior axis

Retinotectal circuits for object size tuning are non-uniformly distributed along the anterior-posterior (A-P) axis of the tectum.^20^^,^^21^ Anterior tectal cells tend to respond more frequently to whole-field motion and large objects, whereas cells responding to local motion and small dot stimuli are biased to the medial to posterior tectum. To test whether the observed shift in tectal population response is locally confined, we classified tectal cell bodies along the A-P axis (Figure 3E). This analysis confirmed that medial and posterior tectal neurons were preferentially tuned to smaller size visual dots compared to anterior neurons. Strikingly, we observed that this topographic preference along the A-P axis was nullified post-MTII treatment and was comparable to responses observed in fed larvae (Figures 3F–3I). Analysis of the actual numbers of activated tectal cells further revealed that this shift is attributed to a loss of neurons responding to small but a gain of neurons responding to large stimuli, in particular in medial-to-posterior tectal regions of sated larvae and starved larvae treated with the Mc4r agonist (Figure S7).

### Loss of melanocortin signaling in tectal cells promotes avoidance behavior toward small-sized stimuli

**Figure 4:**
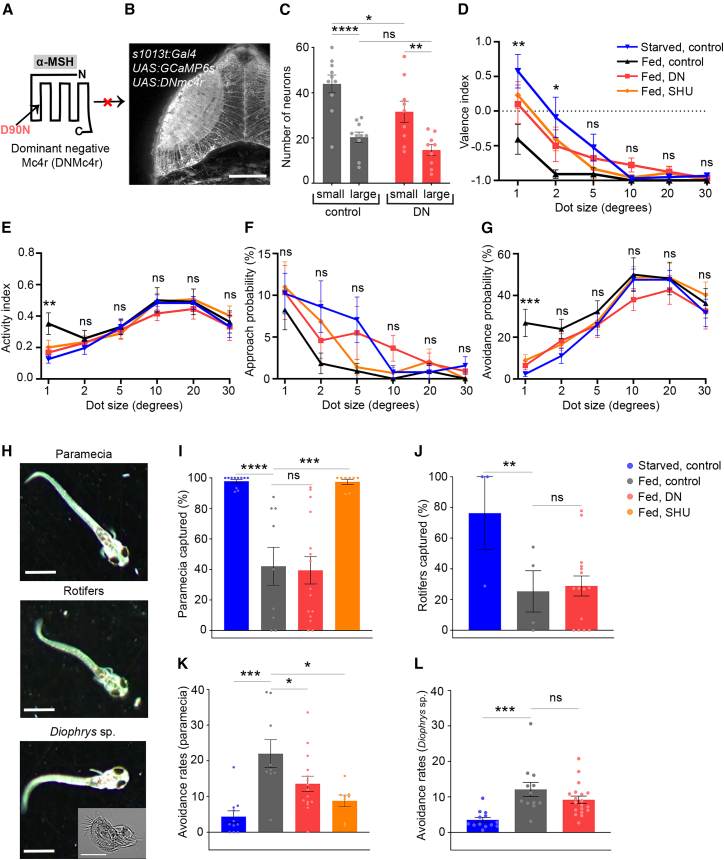
Interference with the tectal melanocortin system affects decision to avoid small-sized stimuli (A) Schematic of dominant-negative Mc4r (DNMc4r) with a D90N amino acid exchange. Figure adapted with permission from Reinoss et al.^8^ (B) Dorsal-view time projection of a 7 dpf fish used for calcium imaging, expressing *GCaMP6s* and *DNmc4r* (confirmed by genotyping) in the tectum driven by *s1013t:Gal4*. Scale bars, 50 μm. (C) Quantification of small (1–5°) and large (10–30°) size-responsive tectal cells. WT fed (*n* = 10): *s1013t:Gal4 UAS:GCaMP6s*; DN fed (*n* = 9): *s1013t:Gal4 UAS:GCaMP6s UAS:DNmc4r.* (D–G) Dependence of valence (D), activity (E), approach (F), and avoidance (G) probability on dot size in 7 dpf larvae for the following conditions and genotypes: starved and fed *s1013t:Gal4 UAS:GCaMP6s*, fed *s1013t:Gal4 UAS:GCaMP6s UAS:DNmc4r*, fed *s1013t:Gal4 UAS:GCaMP6s* treated with SHU9119. Data presented as mean ± SEM. *n* = 12 to 16 larvae per group. (H) Images representing 7 dpf larvae interacting with paramecia (upper), rotifers (middle), and *Diophrys* sp. (lower, with close-up inset). Scale bars, 2 mm and 50 μm (inset). (I) Successful paramecia uptake events shown as percentage of total paramecia count. *n*_controlstarved_ = 11, *n*_controlfed_ = 9, *n*_s1013t:Gal4:UAS: D__N_m_c4rfed_ = 15, *n*_SHU9119fed_ = 8. (J) Successful rotifer uptake events shown as percentage of total rotifer count. *n*_controlstarved_ = 3, *n*_controlfed_ = 4, *n*_s1013t:Gal4:UAS:D___N__m_c4rfed_ = 15. (K) Avoidance events per unit exposure of paramecia and scaled by 100. *n*_controlstarved_ = 11, *n*_controlfed_ = 9, *n*_s1013t:Gal4:UAS: D___N__m_c4rfed_ = 15, *n*_SHU9119fed_ = 8. (L) Avoidance events per unit exposure to *Diophrys* species and scaled by 100. *n*_controlstarved_ = 13, *n*_controlfed_ = 12, *n*_s1013t:Gal4:UAS: D___N__m_c4rfed_ = 19. ∗*p* < 0.05, ∗∗*p* < 0.01, ∗∗∗*p* < 0.001, ∗∗∗∗*p* < 0.0001; ns, not significant. Two-way ANOVA, followed by Fischer’s LSD test (C), paired Wilcoxon signed-rank test, and Mann-Whitney’s test, was used for independent groups (G and I). *t* test with Benjamini-Hochberg correction. Asterisks indicate statistical significance for control-starved and control-fed comparisons (D–G). Two-way ANOVA followed by Bonferroni’s multiple-comparisons test. Dots represent individual larvae (I–L). Data presented as mean ± SEM. For *p* values, see Table 2. See also Figures S9 and S10; Videos S4, S5, S6, S7, and S8.

To directly assess the functional impact of disrupting melanocortin signaling in tectal neurons, we first examined visually evoked tectal activity after targeted expression of a previously validated dominant-negative Mc4r variant (DNmc4r; Figure 4A).^8^ DNmc4r expression was driven by the *Gal4s1013t line*, which allows transgene expression in tectal cells^22^ (Figure 4B; Video S4). Calcium imaging revealed that loss of Mc4r function in tectal cells did not induce an inverse shift in size tuning. Instead, DNmc4r expression led to an overall attenuation of visually responsive and size-tuned tectal neurons, with a particularly pronounced and statistically significant reduction in responses to small visual stimuli, while responses to larger stimuli were less affected (Figure 4C). These results demonstrate that the DNmc4r construct is functionally effective in tectal neurons and suggest that melanocortin signaling regulates the gain or inhibitory balance of tectal circuits, especially in pathways processing small stimuli, rather than bidirectionally controlling size tuning per se.

Video S4. Expression pattern of s1013t:Gal4, related to Figure 4 Confocal stack of a 7 dpf larva expressing GCaMP6s under control of *s1013t:Gal4.*

Having established the functional efficacy of DNmc4r in tectal cells, we next tested whether tectal or global Mc4r disruption using the antagonist SHU9119 alters size-discriminating behavior. Strikingly, either manipulation shifted behavioral decisions of sated larvae from avoidance to approach of small-sized small objects, resembling the behavior of starved larvae (Figure 4D), whereas the probability to react to the presented visual stimuli was unchanged (Figure 4E). Consistent with our gain-of-function findings (Figure 2), this shift in preference was primarily due to a decreased probability of the larvae to engage in slow avoidance responses toward small-sized objects, resulting in more neutral interactions, while the approach probability was not changed significantly (Figures 4F, 4G, S9, and S10; Table 2). Importantly, we did not observe significant differences in swim velocity or total distance traveled across conditions, indicating that neither tectal nor global Mc4r disruption affected general locomotor activity but rather specifically altered behavioral decision-making (Figure S10).

**Table 2:** Statistical analysis of size-discrimination behavior after genetic or pharmacological disruption of Mc4r signaling.

Figure 4D	Valence index					
Groups compared	Circle size (°)					
Control starved vs. control fed	1	2	5	10	20	30
	0.006	0.013	0.106	0.365	0.387	0.175
Control starved vs. DN fed	0.210	0.280	0.557	0.061	0.420	0.562
Control starved vs. SHU fed	0.281	0.345	0.196	0.801	0.558	0.094

Figure 4E	Activity index					
Groups compared	Circle size (°)					
Control starved vs. control fed	1	2	5	10	20	30
	0.003	0.354	0.989	0.862	0.935	0.770
Control starved vs. DN fed	0.354	0.538	0.810	0.355	0.612	0.997
Control starved vs. SHU fed	0.190	0.513	0.491	0.905	0.774	0.468

Figure 4F	Approach probability					
Groups compared	Circle size (°)					
Control starved vs. control fed	1	2	5	10	20	30
	0.573	0.066	0.057	0.365	0.914	0.187
Control starved vs. DN fed	0.977	0.306	0.709	0.098	0.469	0.652
Control starved vs. SHU fed	0.844	0.653	0.047	0.925	0.475	0.126

Figure 4G	Avoidance probability					
Groups compared	Circle size (°)					
Control starved vs. control fed	1	2	5	10	20	30
	0.0005	0.038	0.405	0.791	0.946	0.638
Control starved vs. DN fed	0.101	0.159	0.983	0.214	0.545	0.943
Control starved vs. SHU fed	0.044	0.251	0.811	0.889	0.908	0.357

Figure S9A	Neutral interaction probability					
Groups compared	Circle size (°)					
Control starved vs. control fed	1	2	5	10	20	30
	0.003	0.354	0.989	0.862	0.935	0.770
Control starved vs. DN fed	0.354	0.538	0.810	0.355	0.612	0.997
Control starved vs. SHU fed	0.190	0.513	0.491	0.905	0.774	0.468

Together, these results emphasize that direct satiety-mediating Mc4r signaling in the tectum modulates the processing of visual stimuli by promoting the behavioral decision to avoid small-sized objects.

### Loss of tectal melanocortin signaling does not affect food uptake

If melanocortin-mediated satiety signaling to the tectum does indeed only affect avoidance versus neutral interactions, with little effect on approaching prey-sized objects (Figure 4), it should have no major impact on food uptake, as both ignoring or turning away from potential prey should prevent feeding. To test this directly, we performed actual feeding assays with live prey of different sizes (paramecia and rotifers; Figure 4H; Videos S5 and S6). In line with former reports for other fish species,^23^^,^^24^ global Mc4r inhibition with SHU9119 increased paramecia consumption in sated larvae (Figure 4I). However, DNmc4r expression in tectal cells did not significantly alter paramecia or rotifer uptake compared to sated non-transgenic controls (Figures 4I and 4J). Together, these findings indicate that while melanocortin signaling in the tectum biases visuomotor decisions by regulating avoidance behavior, the execution of feeding is controlled by additional melanocortin-sensitive circuits outside the tectum.

Video S5. Paramecia uptake by a 7 dpf zebrafish larva, related to Figure 4 Behavior is recorded at 35 frames per second. The movie is shown at the original frame rate (real time).

Video S6. Rotifer uptake by a 7 dpf zebrafish larva, related to Figure 4 Behavior is recorded at 35 frames per second. The movie is shown at the original frame rate (real time).

### Tectal melanocortin signaling promotes active avoidance of free-swimming protozoa in sated larvae

In addition to modulating responses to artificial visual stimuli (Figures 4D–4G), we observed that sated zebrafish larvae actively avoid live, free-swimming protozoa such as paramecia and the slightly smaller ciliate *Diophrys* spec, predominantly through slower turning avoidance swims, which likely incur additional energetic costs (Figures 4H, 4K, and 4L; Videos S7 and S8). In contrast, hungry larvae markedly reduced this avoidance, consistent with a strategy to conserve energy and permit feeding opportunities. Notably, both global blockade of Mc4r signaling and selective inhibition of Mc4r in tectal cells of sated larvae significantly reduced avoidance of free-swimming paramecia, shifting behavior toward the phenotype observed in hungry animals (Figure 4K). Despite this pronounced change in avoidance behavior, actual food uptake in DNmc4r-expressing larvae remained low, as reported earlier. These findings identify tectal melanocortin signaling as a key regulator of active avoidance behavior toward small, free-swimming organisms, independently of feeding execution.

Video S7. Avoidance behavior of a 7 dpf zebrafish larva toward a living paramecium, related to Figure 4 Playback frame rate is reduced by 50%.

Video S8. Avoidance behavior of a 7 dpf zebrafish larva toward a living *Diophrys sp.*, related to Figure 4 Playback frame rate is reduced by 50%.

## Discussion

Our identification of axonal Pomca projections to the zebrafish tectum and potential downstream targets therein (Figure 1) is in line with previously identified hypothalamo-tectal projections in zebrafish^25^ and with formerly reported hypothalamic Pomc projections to Mc4r-expressing cells in the mammalian superior colliculus.^23^^,^^26^^,^^27^^,^^28^^,^^29^^,^^30^ Enhanced representation and perception of food-related visual cues in cortical areas of the brain have been shown in both humans and rodents during states of hunger^31^^,^^32^; however, an involvement of melanocortin signaling at the level of the superior colliculus or optic tectum has not been demonstrated previously.

Here, we show that hypothalamo-tectal melanocortin signaling contributes to the previously reported feeding-state-dependent modulation of size-based approach and avoidance behavior in zebrafish larvae.^3^ Importantly, this is likely one of several mechanisms, as previous work has shown that serotonergic^3^ and dopaminergic^9^ pathways also contribute to feeding-state-dependent modulation of size-discriminating behavior. The changes in the number of neurons activated by small or large objects in anterior and posterior tectal regions of starved larvae after gain of Mc4r signaling (Figures 3 and S7) point to Mc4r-dependent modulation of the excitability of tectal neurons as the underlying mechanism, in line with former findings for other melanocortin target neurons.^8^^,^^33^ In contrast, loss of Mc4r function in tectal cells did not produce an inverse shift in size tuning but instead led to an attenuation of visually evoked tectal activity, especially for small size-responsive cells, while nevertheless promoting prey-oriented behavioral choices by selectively reducing avoidance. This indicates that melanocortin signaling modulates the gain of tectal circuits rather than encoding object size directly. Given that mc4r is expressed in both glutamatergic and GABAergic tectal neurons, reduced activity in inhibitory, size-selective neurons may underlie the diminished avoidance behavior observed upon Mc4r loss of function.

In our behavioral assays, both the energy status as well as interference with Mc4r signaling mainly affected the approach versus avoidance response to small objects mimicking potential prey, whereas the response to larger objects mimicking potential predators was not affected (Figures 2 and 4). This indicates that predators are avoided in any case and independent of the energy status of the animal and its melanocortin system. Consistent with this interpretation, Mc4r signaling primarily affected avoidance of small objects (at the expense of neutral interactions), with comparatively little effect on approach behavior (Figures 4 and S9), suggesting that its effect on actual food or energy uptake is minor, in line with our direct feeding assays (Figure 4). These results suggest that tectal melanocortin signaling biases action selection rather than commanding specific behavioral programs, shifting the probability of avoidance versus neutral interactions depending on the energy state of the animal. A similar interference with avoidance, rather than approach, of prey-like visual stimuli was recently reported for zebrafish mutants lacking Pcp4a, a calmodulin-binding peptide whose expression is down-regulated in sated larvae.^9^

Ecologically, avoidance of even small, prey-sized objects in sated larvae may be adaptive and advantageous. Such behavior could reduce encounters with free-swimming parasites^34^ or minimize mechanical stress to the larvae’s soft, highly vascularized gills, with hunger shifting this risk-benefit balance toward foraging.

Our results indicate that tectal melanocortin signaling does not command specific behaviors but rather biases action selection, selectively modulating active avoidance while leaving feeding execution intact. Consistent with this, blockade of Mc4r in the tectum abolished avoidance of small, free-swimming protozoa in sated larvae, producing behavior similar to that of hungry animals, while actual food intake remained low. Similar principles have been observed in mammals. Klawonn et al.^35^ showed that melanocortin signaling can bias behavioral choices, including avoidance of aversive stimuli, without directly altering feeding, suggesting a conserved role of melanocortin pathways in regulating energy-dependent behavioral strategies independently of ingestion.

Together, our study identifies a previously unrecognized role of hypothalamic melanocortin signaling in shaping energy-dependent behavioral decisions in larval zebrafish. Specifically, melanocortin signaling in the tectum biases avoidance of small visual stimuli based on internal energy state, without directly affecting prey consumption. These findings reveal that the melanocortin system modulates sensorimotor processing to allocate behavioral energy expenditure, highlighting a mechanism by which internal state selectively tunes action selection rather than commanding specific behaviors. This work expands the functional repertoire of melanocortin signaling beyond feeding regulation to include energy-dependent visuomotor control.

### Limitations of the study

Although we manipulated Mc4r signaling genetically and pharmacologically, the precise synaptic and circuit-level mechanisms underlying tectal modulation remain to be fully elucidated. In addition, the Gal4 line used for disrupting Mc4r signaling is not entirely tectum specific: it drives expression in some pallial cells of the telencephalon and very sparsely in the hindbrain. Therefore, we cannot rule out that melanocortin signaling also contributes to behavioral modulation in these other regions. Finally, behavioral assays were limited to visual size discrimination and avoidance; other sensory modalities or more complex ecological contexts were not assessed. For instance, future studies have to demonstrate that tectum-driven avoidance of small swimming objects can indeed protect the fish from damage.

## Resource availability

### Lead contact

Further information and requests for resources and reagents should be directed to and will be fulfilled by the lead contact, Dominique Förster (d.foerster@uni-koeln.de).

### Materials availability

This study did not generate new unique reagents.

### Data and code availability

• All data reported in this paper will be shared by the lead contact upon request.
• All original code has been deposited at Zenodo and is publicly available at https://doi.org/10.5281/zenodo.20015261 as of the date of publication.
• Any additional information required to reanalyze the data reported in this paper is available from the lead contact upon request.

## Acknowledgments

We thank Carina Thomas for assistance with experiments. We are especially grateful to Nicholas Guilbeault and Tod Thiele for their support and guidance with the BonZeb behavioral analysis platform. Work in M.H.’s laboratory was supported by the Deutsche Forschungsgemeinschaft (DFG, German Research Foundation) via its Research Training Group GRK 1960, project number 233886668, and by the US National Institute of General Medical Sciences (GM63904). Work in D.F.’s laboratory was supported by DFG Research Grant 544966926, and work in A.A.’s laboratory was supported by the DFG through the Special Priority Programme: “SPP 2205 Evolutionary optimization of neural processing” (430158665; AR 1076/1-2, AR 1076/1-1), by the Major Research Instrumentation Program of the DFG (INST 37/1254-1 FUGG), and by the Human Frontier Science Program (HFSP) Young Investigator grant RGY0079.

## Author contributions

M.P., D.F., and M.H. conceived the project. M.P. performed all experiments, assisted by F.S., L.v.G.J., and K.B. for the behavioral experiments and by T.H., Y.Z., and A.A. for the Ca-imaging experiments. S.L. helped with analysis of behavioral data. M.P., D.F., and M.H. wrote the manuscript.

## Declaration of interests

F.S. is cofounder, shareholder, and CEO of ariadne.ai AG.

## STAR★Methods

### Key resources table

**Table:** 

REAGENT or RESOURCE	SOURCE	IDENTIFIER
**Antibodies**		

Chicken anti-GFP	Thermo Fisher	Cat # A10262; RRID: AB_2534023
Rabbit anti-RFP	MBL International	Cat # PM005; RRID: AB_591279
Mouse Anti-SV2	DSHB	RRID: AB_2315387
Donkey anti-rabbit Alexa 488	Thermo Fisher	Cat# A21206
Goat anti-chicken Alexa 488	Thermo Fisher	Cat# A11039; RRID: AB_142924
Goat anti-rabbit Alexa 555	Thermo Fisher	Cat# A21428; RRID: AB_141784
Goat anti-mouse Alexa 647	Thermo Fisher	Cat# A21235; RRID: AB_2535804

**Chemicals, peptides, and recombinant proteins**		

Melanotan II acetate salt	Sigma	Product# M8693
SHU 9119	Phoenix Pharmaceuticals	Cat# 043-24
Proteinase K	Genaxxon	Article# M3036.0500
DAPI	Thermo Fisher	Cat# D1306
Glycerol	Carl Roth	Article# 3783.2
Dulbecoo’s PBS (with calcium and magnesium salts)	Sigma	Product# D8662
1X Dulbecoo’s PBS (without calcium and magnesium)	GIBCO, Thermo Fisher	Cat# 14190144
Paraformaldehyde (PFA)	Sigma	Product# P6148
Tween 20	Sigma	Product #P1379

**Critical commercial assays**		

HCR^TM^ RNA-FISH (v3.0) reagents	Molecular Instruments	https://www.molecularinstruments.com/

**Experimental models: Organisms/strains**		

Zebrafish: TgBAC(pomca:EGFPras)^fr38Tg^	Löhr et al.^10^	ZFIN: ZDB-ALT-191209-2
Zebrafish: Tg(Et(−1.5hsp70L:Gal4-VP16)^s1013t^	Scott et al.^22^	ZFIN: ZDB-ALT-070420-14
Zebrafish: Tg(-7atoh7:Gal4-VP16)^s1992t^	Del Bene et al.^36^	ZFIN: ZDB-ALT-110912-2
Zebrafish: Tg(UAS:NpHR-mCherry)^s1989t^	Arrenberg et al.^37^	ZFIN: ZDB-ALT-101227-3
Zebrafish: Tg(5xUAS-hsp70L:GCaMP6s)	Muto et al.^38^	ZFIN: ZDB-ALT-170615-4
Zebrafish: TgBAC(pomca:KALTA4)^fr39Tg^	Löhr et al.^10^	ZFIN: ZDB-ALT-180904-1
Zebrafish: Tg(UAS:Tomato-CAAX)^rw0315Tg^	Miyasaka et al.^39^	ZFIN: ZDB-TGCONSTRCT-160411-2
Zebrafish: Tg(UAS:Syp-GFP)^ic3021Tg^	Gebhardt et al.^40^	ZFIN: ZDB-ALT-150601-2
Zebrafish: Tg(elavl3:H2B-GCaMP6f)^jf7Tg^	Quirin et al.^41^	ZFIN: ZDB-ALT-150916-4
Zebrafish: Tg(UAS:DNmc4r)^fr29Tg^	Reinoss et al.^8^	N/A

**Oligonucleotides**		

Primer: genotyping Gal4 forward CCAAAAGGTCTCCGCTGACTA	IDT	This paper
Primer: genotyping Gal4 reverse CAGTCTCCACTGAAGCCAATC	IDT	This paper
Primer: genotyping 5xUAS forward GACGGTATCGATAAGCTT	IDT	This paper
Primer: genotyping zmc4r reverse CATGGTGAAGAACATGCT	IDT	This paper

**Software and algorithms**		

MATLAB R2022a	Mathworks	https://www.mathworks.com/products/matlab.html
Fiji 1.54i	NIH	https://imagej.net/ij/
Prism Version 10.5.0	GraphPad Software	https://www.graphpad.com
Psychopy	Open Science Tools Ltd. Peirce^42^	https://www.psychopy.org
Mscan	Sutter Instrument	https://www.sutter.com/microscopes/mom
Python version 3.8 used for Pyschopy	Python Software Foundation	https://www.python.org
Python code for Size discrimination assay	This paper	https://doi.org/10.5281/zenodo.20015261
Code for visual stimulation for calcium imaging	VxPy Version 0.14	Soto et al.^18^ PREPRINT
Python code for calcium imaging analysis	N/A	Zhang et al.^43^

**Other**		

8-inch monitor, Resolution: 1024 × 768	Beetronics	Model: 8VG3
Basler acA2440-75uc USB 3.0 camera	Basler AG	https://www.baslerweb.com/en/

### Experimental model and study participant details

#### Experimental zebrafish models

All animal experiments were approved by the regional animal care committees (2024-410-Grundantrag). Zebrafish were raised under standard conditions at 28°C on a 14 h light/10 h dark cycle. Larvae were raised in E3 medium and fed from 5 dpf onward with paramecia. For experiments involving fasting, food was removed six days post-fertilization (dpf) and experiments were performed the following day (7 dpf). All experiments were performed at 7 dpf (standard length = 3.5–4.0 mm). At this stage, the sex of the fish is not fully specified yet, and hence animals were randomly allotted to experimental groups. The following previously published transgenic lines were used in this study: *TgBAC(pomca:EGFPras)*^*fr38Tg*^,^10^ *Et(−1.5hsp70L:Gal4-VP16)*^*s1013t*^,^22^ *Tg(-7atoh7:Gal4-VP16)*^*s1992t*^,^36^ *Tg(UAS:NpHR-mCherry)*^*s1989t*^,^37^ *Tg(5xUAS hsp70L:GCaMP6s)*,^38^ *TgBAC(pomca: KALTA4)*^*fr39Tg*^,^10^ *Tg(UAS:Tomato-CAAX)*^*rw0315Tg*^,^39^ *Tg(UAS:sypb-GFP)*^*ic3021Tg*^,^40^ *Tg(elavl3:H2B-GCaMP6f)*^*jf7Tg*^,^41^ *Tg(UAS:DNmc4r)* ^*fr29Tg*^.^8^ Carriers of the *Tg(UAS:DNmc4r)*^*fr29Tg*^ transgene were identified by PCR using primers 5′-GACGGTATCGATAAGCTT3′ and 3′- CATGGTGAAGAACATGCT- 5′ for DNmc4r region, and 5′- CCAAAAGGTCTCCGCTGACTA-3′ and 3′- CAGTCTCCACTGAAGCCAATCT-5′ for Gal4 region.

### Method details

#### Whole mount immunofluorescence imaging and image processing

For whole-mount immunofluorescence (IF) larvae were fixed at 7 dpf in 4% PFA overnight at 4°C with gentle shaking. Post fixation samples were washed thrice in PBST (PBS with 0.3% Tween), the gut and jaw were dissected out and larvae were stored in 100% methanol at −20°C until further used. For Figure 1C, the eyes were dissected out for visualization of the optic chiasm and RGC axons. On the day of the experiment, after performing gradient rehydration (75%-50%–25%) with methanol in PBST, larvae were digested with 1 mL Proteinase K (10 μg/mL) for 60min at room temperature. Following digestion, larvae were blocked in blocking solution (10% Fetal Calf Serum in PBST) for at least 2 h at room temperature and then incubated in primary antibody dissolved in blocking solution overnight at 4°C. Primary antibodies against GFP (Thermo Fischer, Cat#A10262, 1:800), MBL International, Cat#PM005,1:800), and SV2 (DSHB, AB_2315387, 1:500) were used. The following day, samples were washed with PBST and incubated in Alexa Fluor secondary antibodies (1:800) anti-chicken-Alexa488, anti-rabbit-Alexa555, and anti-mouse-Alexa647 along with nuclear stain DAPI at 4°C. Post antibody incubation larvae were washed with PBST and stored in 80% glycerol in PBS until used for imaging. After mounting in 100% glycerol, larvae were imaged using a Zeiss LSM700 or LSM710 confocal microscope.

Image registrations of larvae expressing *atoh7:Gal4 UAS:NpHR-mCherry pomca:EGFPras* were performed using ANTs. One image stack of mCherry expression was selected as a reference, and additional mCherry stacks were co-registered using the following ANTs registration command:

antsRegistration -d 3 --float 1 -o [${output1},${output2}] --interpolation WelchWindowedSinc --use-histogram-matching 0 -r.

[${template},${input1},1] -t rigid[0.1] -m MI[${template},${input.

1},1,32,Regular,0.25] -c [200 × 200×200 × 0,1e−8,10] --shrink-factors.

12 × 8×4 × 2 --smoothing-sigmas 4 × 3×2×1vox -t Affine[0.1] -m MI[${tem-

plate},${input1},1,32,Regular,0.25] -c [200 × 200×200 × 0,1e−8,10]

--shrink-factors 12 × 8×4 × 2 --smoothing-sigmas 4 × 3×2 × 1 -t SyN[0.01,6,0.0]

-m CC[${template},${input1},1,2] -c [200 × 200×200 × 200×10,1e−7,10]

--shrink- factors 12 × 8×4 × 2×1 --smoothing-sigmas 4 × 3×2 × 1×0.

Afterward, the following transformation command was applied to the EGFP channel:

antsApplyTransforms -d 3 -v 0 --float -n WelchWindowedSinc

-i ${input3} -r ${template} -o ${output4} -t ${output1}1Warp.nii.gz.

-t ${output1}0GenericAffine.mat.

Intensity profiles of *pomca:EGFPras* expression in the tectal neuropil were generated in Fiji as previously described.^25^

#### *In situ* hybridization

*In-situ* hybridization to detect *mc4r, gad1b, vglut2a, pomca* and *agrp* transcripts were performed using third generation *in situ* HCR v3.0. All the components, reagents, probes, hairpins and required buffers were purchased from Molecular Instruments. *In-situ* HCR in wholemount zebrafish larvae (nacre −/−, 7 dpf) was carried out by adapting protocol described.^44^ Briefly, larvae were fixed overnight in 4% PFA at 4°C with gentle shaking. Post fixation larvae were washed with Dulbecoo’s PBS (PBS without calcium and magnesium and 0.1% Tween 20) DPBST. Samples were stored in 100% methanol at −20°C to dehyrdate and permeabilize the tissue. Prior to the experiment the gut and jaw of larvae were removed for easy penetration of the probes. Approximately ten larvae were transferred to a 2 mL Ependorff tube and serial rehydration was performed by washing in 50% MeOH/50% PBST and 25% MeOH/75% PBST for 5 min each and lastly washed in PBST for five times, 5 min each. Samples were pre-hybridized with probe hybridization buffer for 30min at 37°C with gentle shaking. Probe solutions were prepared by transferring 2 pmol of each HCR probe set (2 μL of 1 μM stock) to 250 μL of hybridization buffer at 37°C. For *mc4r* colocalization with *gad1b* and *vlgut2a*, 4 μL of each probe from 1 μM stock was used. The hybridization buffer was replaced with probe solution, and samples were incubated for 12 to 16 h at 37°C with gentle shaking. Subsequently, excess probe was removed by washing in pre-warmed probe wash buffer 4 × 15 min at 37°C with gentle shaking. Next, larvae were washed for 2 × 5 min with 5X SSCT (5X sodium chloride sodium citrate +0.1% Tween 20) at room temperature and pre-amplification was performed by incubating samples in 250 μL of amplification buffer for 30 min at room temperature. Meanwhile, 30 pmol of hairpin h1 and 30 pmol of hairpin h2 were prepared separately by snap-cooling 5 μL of 3 μM stock by incubating the hairpins in 95°C for 90 s and cooling down at room temperature in dark for 30 min. After cooling, hairpin solution was prepared by transferring h1 and h2 hairpins to 250 μL amplification buffer. The pre-amplification buffer was removed, and samples were transferred to hairpin solution for 12 to 16 h in the dark at room temperature. Next day, excess hairpins were washed 3 × 20 min using 5X SSCT at room temperature with gentle shaking. For nuclear staining larvae were incubated overnight with DAPI in 5X SSCT at 4°C. Subsequently, larvae were washed briefly with 5X SSCT followed by a single PBST wash and stored in 80% glycerol at 4°C. All samples were imaged on a Zeiss LSM700 confocal microscope within one week.

For quantifying the changes in the expression levels of *agrp1* and *pomca* genes in starved and fed larvae, the confocal parameters - laser power, gain, magnification and dimensions of the imaging frame were kept consistent across all imaging sessions. Fluorescence intensity was quantified in ImageJ by manually selecting cells using the elliptical selection tool and recording the mean gray value for each cell. In addition, the centroid (X, Y) coordinates of the selected cells were extracted to enable spatial mapping along the rostral-caudal axis of the hypothalamus. For each gene, fluorescence intensities from both starved and fed were pooled to identify the global minimum and maximum mean gray values. These values were subsequently used to normalize intensity measurements from individual feeding states, generating a normalized expression range from 0 to 1, where 1 represents the highest observed fluorescence intensity. This enabled direct comparison of expression profiles across conditions. The centroid coordinates of individual cells were used to map normalized fluorescence intensities along rostral-caudal axis of the hypothalamus onto an anatomical reference in MATLAB. DAPI co-staining acquired during HCR was used as the anatomical reference for alignment.

#### Size discrimination assay - feeding protocol and pharmacological treatments

For the paradigm described in Figure 2A, zebrafish larvae (Tüpfel long-fin, TL) were fed from 5 dpf with paramecia in petri dishes with Danieau’s solution (58 mM NaCl, 0.7 mM KCl, 0.4 mM MgSO_4_, 0.6 mM Ca(NO_3_)_2_, 5.0 mM HEPES pH 7.6). On 6 dpf, larvae were transferred to a six-well plate with Danieau’s solution with five larvae in each well. The larvae in the fed group were provided with paramecia, while the starved group received no food. On 7 dpf, both the groups received a fresh change in the medium and some wells were treated with freshly prepared drug solutions Melanotan II (MTII, Sigma, 10 μM working concentration) prepared in Danieau’s solution for 3–4 h prior to the recordings. Simultaneously, control groups received a change in medium (in starved group) and food (in fed group). Before the treatment, freshly prepared solutions were blinded by an investigator. Prior to the recordings, both the groups received a fresh change in medium. Subsequently, larvae were transferred to the recording chamber with Danieau’s solution. For behavior experiments performed in transgenic larvae (related to Figure 4), *Tg(s1013t:Gal4, UAS:DNmc4r)* fish were crossed to *Tg(UAS:GCaMP6s)*, to allow sorting for strong expressors and were then used for behavior experiments. To ensure blinded experiments, fish were genotyped for the presence of *UAS:DNmc4r* after the larva-dot interactions were scored, and subsequently individual identities of larvae used for experiments were revealed for group comparisons. Simultaneously in a control experiment, larvae obtained from *Tg(s1013t:Gal4)* crossed to *Tg(UAS:GCaMP6s)* were treated with SHU9119 (SHU, Phoenix Pharmaceuticals, 20 μM working concentration).

#### Size discrimination assay - Behavioral recordings and analysis

Size discrimination assay was performed as described previously.^3^^,^^15^ A single larva was placed in a custom-made transparent plastic chamber (15.6 × 2.6 cm) containing Danieau’s solution placed on a computer screen (8-inch) displaying moving black circles on a white background with a constant speed of 42°/s. The distance between each dot stimulus that was presented was 4 cm. Visual stimuli were generated using PyschoPy3 and a custom written Python script. The thickness of the plastic chamber bottom was 4 mm and there was no air gap between chamber and screen. The vertical distance between the screen and the water surface was 15.8 mm, and this value was used in the PsychoPy (“monitor center”) to calculate the visual angle occupied by the circle when positioned beneath the larva. The resulting degrees of visual field ranged from 1° to 50°. Since the larva was freely moving, the exact dot size at the position of interaction is an approximate estimation (it might have appeared larger to the larva, when it was swimming at the bottom of the dish). The reported size of each dot is used as a means for distinguishing between relative sizes of the visual stimuli as described (Filosa et al., 2016).^3^ Each dot was presented nine times to avoid habituation to the stimulus presented and reduction in the efficacy of the drug post treatment. The level of water in the behavior chamber was kept consistent and after the larva were placed in the recording chamber, the lights were turned off, to ensure the visual stimulation monitor was the only source of light. Room temperature was maintained between 26°C and 28°C. Videos were recorded with a camera positioned above the chamber at 35 fps using a Basler Camera and software (Basler AG, Germany). Larva-stimuli interaction was scored as an approach or an avoidance when the fish swam toward (including slow forward swims and J-turns) or away from a moving circle, respectively. Neutral interaction was scored when fish swam neither toward or away from the stimuli.^15^ Interactions were initially scored manually in a double-blind manner by two independent experimenters and subsequently confirmed using automated behavioral analysis with the BonZeb platform.^45^ This automated analysis allowed objective and reproducible quantification of behaviors and enabled differentiation between slower avoidance maneuvers, relevant for interactions with small objects, and fast escape-like responses, typically elicited by larger stimuli, based on swim velocity and distance moved during avoidance.

To quantify the preference of fish to approach or avoid visual stimuli, a valence index [(approaches – avoidances)/(approaches + avoidances)] was used. Efficiency of larva-dot interactions was quantified by an activity index [(approaches + avoidances)/(approaches + avoidances + neutral interactions)].^3^^,^^15^ Approach, avoidance and neutral interaction probabilities were calculated as [approaches/(approaches + avoidances + neutral interactions)] or [avoidances/(approaches + avoidances + neutral interactions)] or [neutral interactions/(approaches + avoidances + neutral interactions)], respectively.^9^ The identity of the groups was only revealed after the analysis was performed. For performing size discrimination assay in transgenic DNmc4r larvae, the genotype of the larvae was revealed only after performing the recordings and analysis.

#### *In vivo* two-photon calcium imaging

For the experiment described in Figure 3, activity of tectal neurons was recorded in 7 dpf *Tg(elavl3-H2B-GCaMP6f)* larva embedded in low melting agarose (1.6% in E3 medium) using a spherical glass container previously described.^19^ Prior to the experiment, all larvae were fed with paramecia at 5 dpf. On day 6, food was removed in starved group, and fed group received paramecia. On the day of experiment (7 dpf), both the groups received a change in embryo medium (E3) and only the fed group received paramecia. Embedding of larvae in agarose for imaging was followed as described previously.^18^^,^^43^ Briefly, a single fish was embedded in low melting agarose (1.6%) prepared in 1X E3 medium.^46^ The agarose surrounding the fish head was kept trimmed on the sides and in front of the animal. Mounted fish (Figure S5) was then carefully transferred to the center of a spherical glass-bulb container filled with transparent E3 medium and held stably using a shaft holding to the back of the stage (Figure S5). The body position and orientation of the mounted fish were adjusted by rotating the shaft, the fish was centered with dorsal side facing up and the nose pointing straight to the front without any tilting. Visual stimuli of different sizes (2°–30° visual angle) were presented on a spherical glass container as bright dots against a dark background using a custom-written algorithm (Figure S5). Each stimulus of the same size was presented for six repetitions, moving in alternating clockwise and anti-clockwise directions from one side (−90° azimuth) to the other side (+90°) and back at a speed of 40°/s. In each repetition, every 9 s stimulus phase was preceded by a 5 s pause phase, during which the dot stopped moving. Calcium imaging began 15 s before, and ended 15 s after stimulus presentation.

For experiments involving MTII treatment in starved larvae, the first session of recordings was performed prior to the drug treatment. After the first session, the stage with the embedded larvae was removed from the glass container and placed in a Petri dish with MTII (Sigma, 10 μM) dissolved in E3 medium for 3–4 h. The treated larva was placed back in the spherical-glass container for a second session of post-treatment recordings. Untreated fed control larvae were embedded and placed in E3 medium for the same duration as the drug treated larvae and used directly for recordings. Calcium imaging was performed with a two-photon microscopy set up (Sutter Instruments, Novato, California, USA) coupled to a Coherent Vision-S Ti-Sa laser. The emitted fluorescence light of the calcium indicator protein, GCaMP6f, triggered by the excitation laser beam at 920 nm wavelength was acquired with a 20x/1.0 Zeiss objective. The image time series was recorded using MScan software (Sutter instruments). The acquisition frame rate was 2 fps at 512 × 512 pixels and magnification of 2 (0.44 × 0.44 μm^2^ per pixel). Responses to visual stimuli were recorded from one hemisphere of the tectum as shown in Figures 3A and 3B. Along the z-plane, the pretectum region (at the level of the posterior commissure) was selected as a landmark and position of this plane was set as “0 μm position”. Recordings in the tectum were performed by selecting different dorsoventral planes, starting with the ones close to reference point, and gradually moving upwards toward the dorsal end with a step size of 5–10 μm. Tectal planes recorded prior to the drug treatment could be roughly traced back post-drug treatment by identifying the neuronal somata localized in the neuropil of the tectum.

#### Processing and analysis of calcium imaging data

All the recordings were processed for motion artifacts along XY plane by a phase-correlation algorithm, and visible cells in the tectal periventricular region on a time-averaged image of the corrected video were selected as region of interests (ROIs) by using a marker-controlled watershed algorithm as described.^43^ Segmentation of ROIs for the transgenic fish *Tg(s1013t:Gal4, UAS:DNmc4r UAS:GCaMP6s)* was performed manually by observing the calcium trace using the algorithm mentioned above. Responses from non-neuronal cells (such as glia) were not used for analysis. The coordinate (x,y) positions of the selected ROIs were used to segregate the ROIs along the anterior-posterior axis of the tectum. For each ROI, the calcium time series was extracted as the sum of all pixel values within the ROI for each frame. From these ROI traces, ΔF/F values were calculated as (F_t_-F_0_)/F_0_, where F_t_ is the fluorescence at time t and F_0_ is the baseline fluorescence, defined as average fluorescence during the 15 s pause phase preceding the first stimulus presentation. To reduce noise interference, ΔF/F fluorescence traces were smoothened using a sliding median filter (the median of three data points), and a selection threshold was applied to identify neurons having a ΔF/F pixel value of at least 0.5 or greater across time frames. This allowed eliminating neurons that were not responsive to any of the phase stimulations. Normalized ΔF/F responses were averaged across all repetitions (time-averaged ΔF/F signal during 9 s stimulus phase without deconvolution) and weighted mean response (WMR) angles for individual neurons were calculated with modifications to the previously described.^3^ Briefly, response weights (w_i_) for each stimulus i were calculated by taking response of a neuron for each stimulus over the sum of all n responses to the visual stimuli. These weights were then multiplied with the respective logarithmic (log_10_) angular sizes of visual stimuli (x_i_) and weighted sums of visual stimulus sizes was calculated by following equation:

$$\log\left({WMR}_{angle}\right)=\sum_{i=1}^{n}w_{i}\times \log\left(x_{i}\right)$$

Where, weights (wi) correspond to:

$$w_{i}=\frac{\left(\Delta F/F\right)i}{\sum_{i=1}^{n}\left(\Delta F/F\right)i}$$

The WMR angles for each group (Figure 3), were plotted by taking cumulative frequency percentage distribution in GraphPad Prism.

The position of neurons shown in the cumulative frequency plots for each group was masked onto a mask containing the coordinate positions of the selected ROIs. Subsequently, WMRangles were segregated along anterior-posterior axis of the tectum by drawing a vector scale from 1 to 10 along the diagonal length of the neuropil of the tectum in MATLAB (as indicated in Figure 3E). Neurons falling within the scale of 1–3, 4–6 and 7–10 were considered as anterior, medial, posterior respectively. Average of WMR angles obtained for respective regions were used for the bar graph. ΔWMRangles were calculated by taking the absolute difference between WMR angles before and after MTII treatment for each region.

#### Food uptake assay

Food uptake assay was performed on transgenic fish *Tg(s1013t:Gal4, UAS:DNmc4r UAS:GCaMP6s)* crossed to wildtype TL. Prior to the experiment, larvae were sorted for the presence of strong GCaMP expressors in the tectum at 4 dpf. Feeding paradigm used for this assay was similar to the paradigm used for size discrimination assay. Briefly, all animals were fed with paramecia from 5 dpf. On day 6, the larvae in starved group were deprived of paramecia, while the fed group received paramecia. The assay was performed on 7 dpf larvae. Prior to the assay, fed group received paramecia, and starved group received change of solution (Danieau’s solution). The assay was performed in a recording chamber (diameter: 20 mm; depth: 2.5 mm; CoverWell Imaging Chambers Grace Bio-Labs) placed on a custom-built acrylic holder. The recording chamber was illuminated from below using a ring LED light (Schott KL 1500 LCD) as described.^38^ Single larvae were placed in the chamber. After 15 min of acclimatization, paramecia or rotifers were introduced and the chamber was closed with a cover glass. Paramecia culture used for the assay was first filtered using a 100-micron sieve to remove large unwanted debris and later passed through a 20-micron sieve. The paramecia retained on the filter was washed into a clean Petri dish with Danieau’s solution. Rotifers (Brachionus plicatilis) used for the assay were acclimatized to low salinity (5 ppt) one-day prior to the experiment at room temperature. The final salinity of the solution used for the assay was approximately 2 ppt. Video recordings were performed using Basler camera and software at 35 fps. After the recordings, the number of paramecia or rotifer uptakes were counted manually. Other ciliates (*Diophrys* species) were manually picked from the in-house growing paramecia cultures and used to quantify fish avoidance rates in behavioral assays.

To ensure blinded experiments, fish were genotyped for the presence of *UAS:DNmc4r* after the uptake events were scored, and subsequently individual identities of larvae used for experiments were revealed. Simultaneously in a control experiment, larvae obtained from *Tg(s1013t:Gal4)* crossed to *Tg(UAS:GCaMP6s)* were treated with SHU9119 (SHU, Phoenix Pharmaceuticals, 20 μM working concentration).

### Quantification and statistical analysis

Statistical analysis was performed using GraphPad Prism (version 10.6.1). Significance was determined by Wilcoxon-matched paired signed rank test for paired groups; Mann Whitney’s test for comparing unpaired non-parametric datasets; two-way ANOVA for multiple comparisons corrected using Tukey’s statistical hypothesis testing and multiple *t* test along with Benjamini-Hochberg method for controlling false discovery rate. Data are shown as mean ± SEM where applicable. “n” represents the number of larvae used for each experiment. For plots showing fractions of cells, error bars are not shown. ∗, *p* < 0.05, ∗∗, *p* < 0.01, ∗∗∗, *p* < 0.001; ns, not significant.
