# Supplementary material for: The tectal melanocortin system modulates energy-dependent visual avoidance behavior in zebrafish
Source: iScience. 2026 May 22;29(6):116095. doi: 10.1016/j.isci.2026.116095 (PMC13223957; doi:10.1016/j.isci.2026.116095)
Supplement: Document S1. Figures S1–S10 [file mmc1.pdf]

iScience, Volume *29*

Supplemental information

The tectal melanocortin system modulates energy-dependent visual avoidance behavior in zebrafish

Madhuri Puvvada, Tim Hladnik, Yue Zhang, Fabian Svara, Silke Lemmens, Louise von Gersdorff Jørgensen, Kevin Briggman, Aristides Arrenberg, Dominique Förster, and Matthias Hammerschmidt

Figure S1. Related to Figure 1

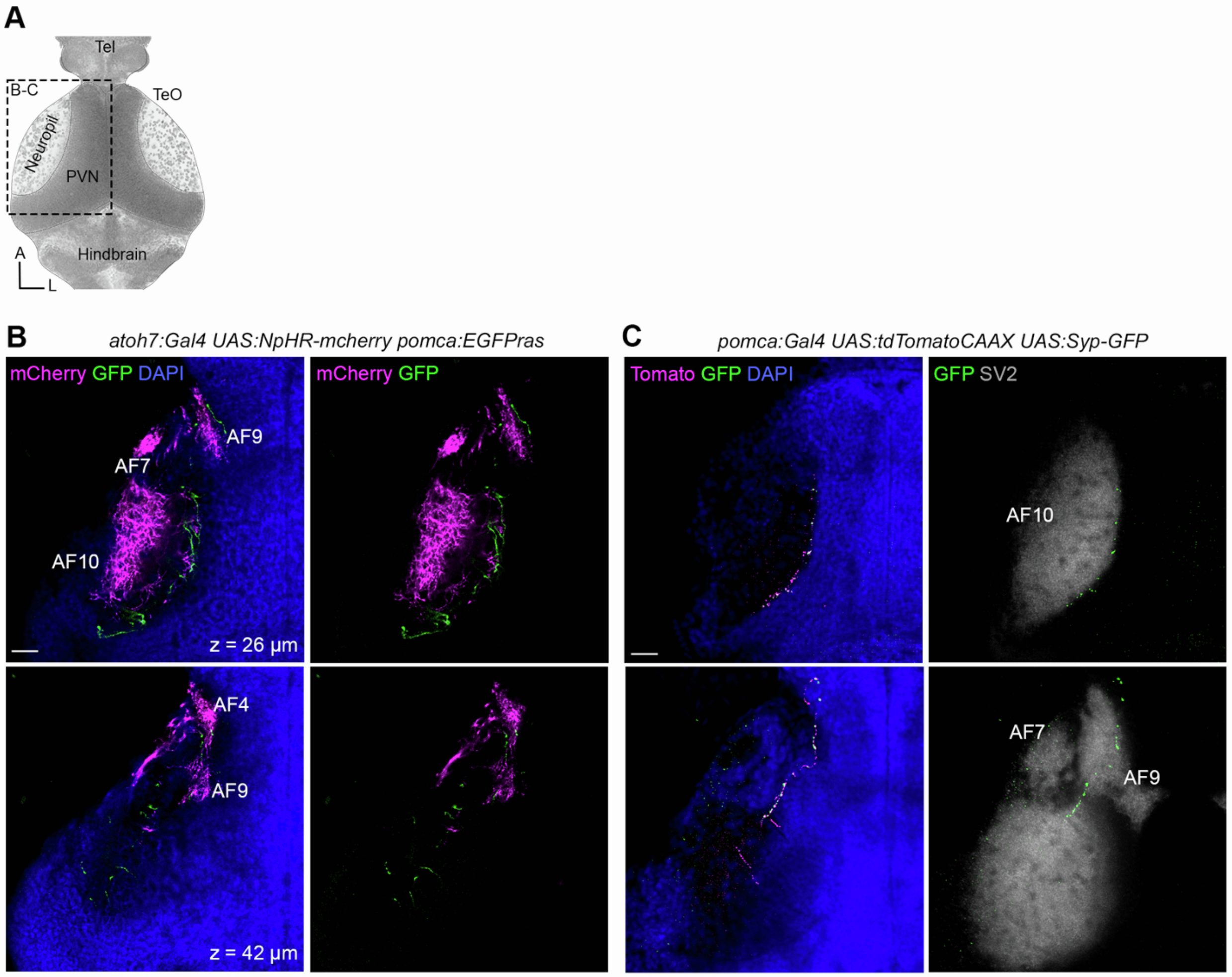

Figure S1. Axonal projections of Pomca neurons in extra-tectal regions of zebrafish larvae. Related to Figure 1.

(A) Schematic illustration of dorsal view of whole-mount zebrafish larvae and depiction of imaged regions.

(B) Immunofluorescence for RFP and GFP on *pomca:EGFPras atoh7:Gal4 UAS:NphR-mcherry* transgenic larvae at 7 dpf. Tectal and extra-tectal arborization fields (AFs) are labeled. Depth of confocal planes is shown in micrometers from the skin. Scale bar, 20 µm, n = 9.

(C) Immunofluorescence for RFP and GFP on *pomca:Gal4 UAS:tdTomatoCAAX* crossed to *UAS:Syp-GFP* transgenic larvae and co-stained with pre-synaptic marker SV2. Tectal and extra-tectal AFs are labeled. Location of the AFs were identified by SV2 staining. Scale bar, 20 µm, n = 4.

Figure S2. Related to Figure 1

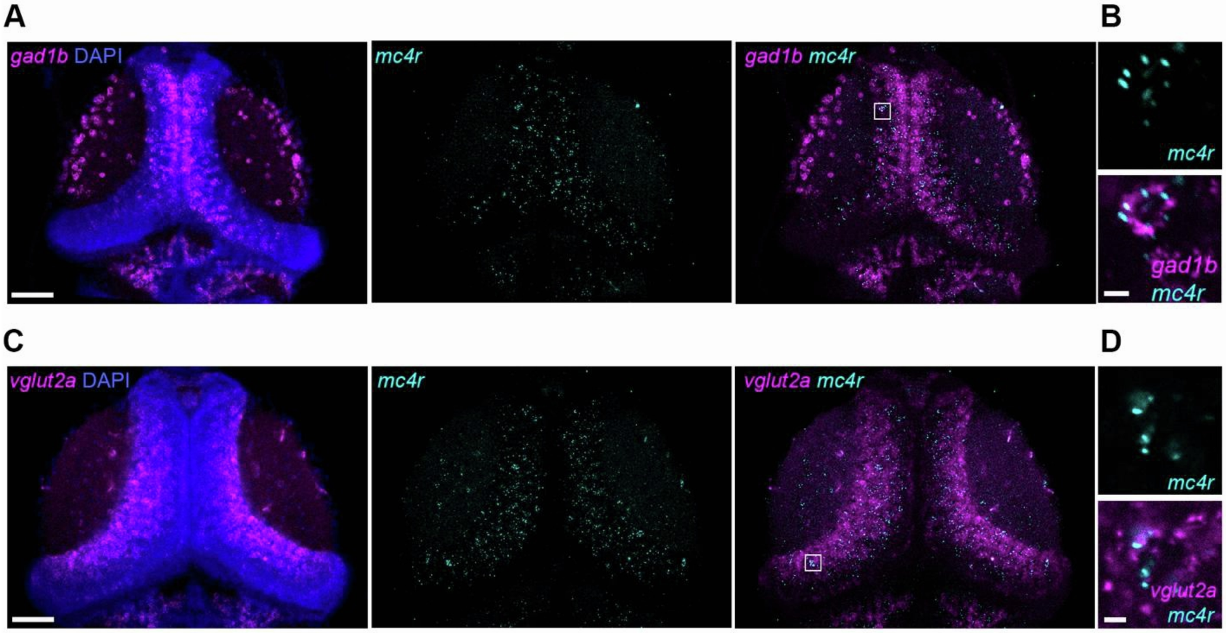

Figure S2. Localization of *mc4r*, *vglut2a* and *gad1b expression* in tectal neurons. Related to figure 1.

(A-B) HCR *in situ* stainings for *mc4r* and *gad1b* in tectal neurons. Scale bar, 50 µm. Close-up (B) of the box highlighted in A shows co-expression of *gad1b* and *mc4r* in a tectal periventricular neuron. Scale bar, 2 µm, n = 6.

(C-D) HCR *in situ* stainings for *mc4r* and *vglut2a* in tectal neurons. Scale bar, 50 µm. Close-up (D) of the box highlighted in C shows co-expression of *vglut2a* and *mc4r* in a tectal periventricular neuron. Scale bar, 2 µm, n = 6.

Figure S3. Related to Figure 2

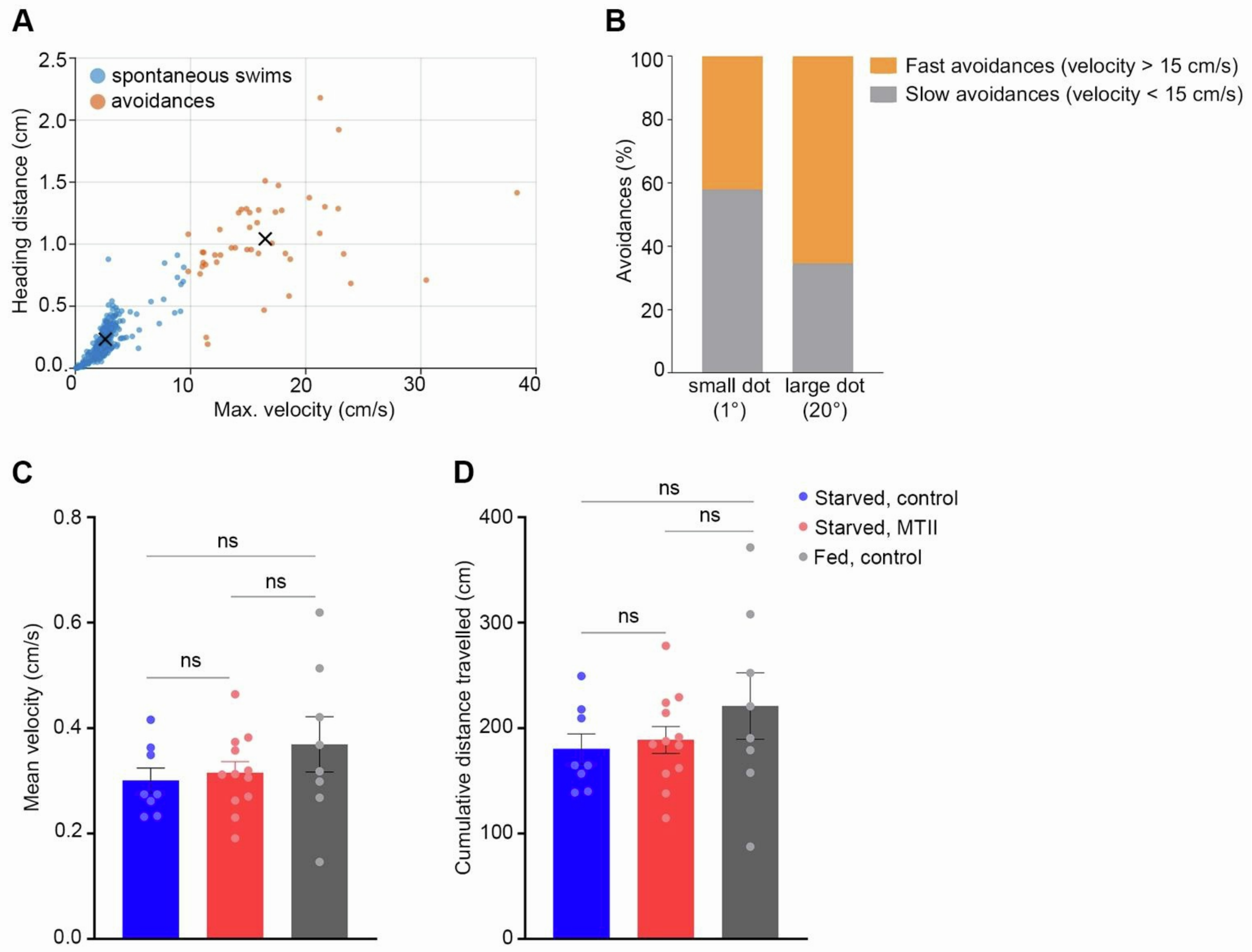

Figure S3. Automated behavior analysis. Related to figure 2.

(A) Swim bouts (n = 386) pooled from presentation of small (1°) and large (20°) stimuli during size-discrimination assay were plotted in velocity-distance space and grouped using unsupervised k-means clustering (*k* = 2). Black crosses denote cluster centroids. Bouts classified as spontaneous swims are colored in blue and avoidance swims in orange, n = 33 fish. (B) Fraction of avoidance events in percentage for bouts classified as avoidance swims in A for small (1°) and large (20°) stimulus sizes. Slow and fast avoidance events were obtained using a velocity threshold less than or greater than 15cm/s, respectively. (C) Mean velocity (cm/s) calculated from centroid tracking over a 10 min recording. $n_{starved,control} = 8$, $n_{fed,control} = 8$, $n_{starved,MTII} = 12$. (D) Cumulative distance travelled (cm) over a 10 min recording. $n_{starved,control} = 8$, $n_{fed,control} = 8$, $n_{starved,MTII} = 12$. Analyzed using Mann-Whitney's (two-tailed) t-test, ns = not signficant (C-D). Bars represent mean ± SEM.

Figure S4. Related to Figure 2

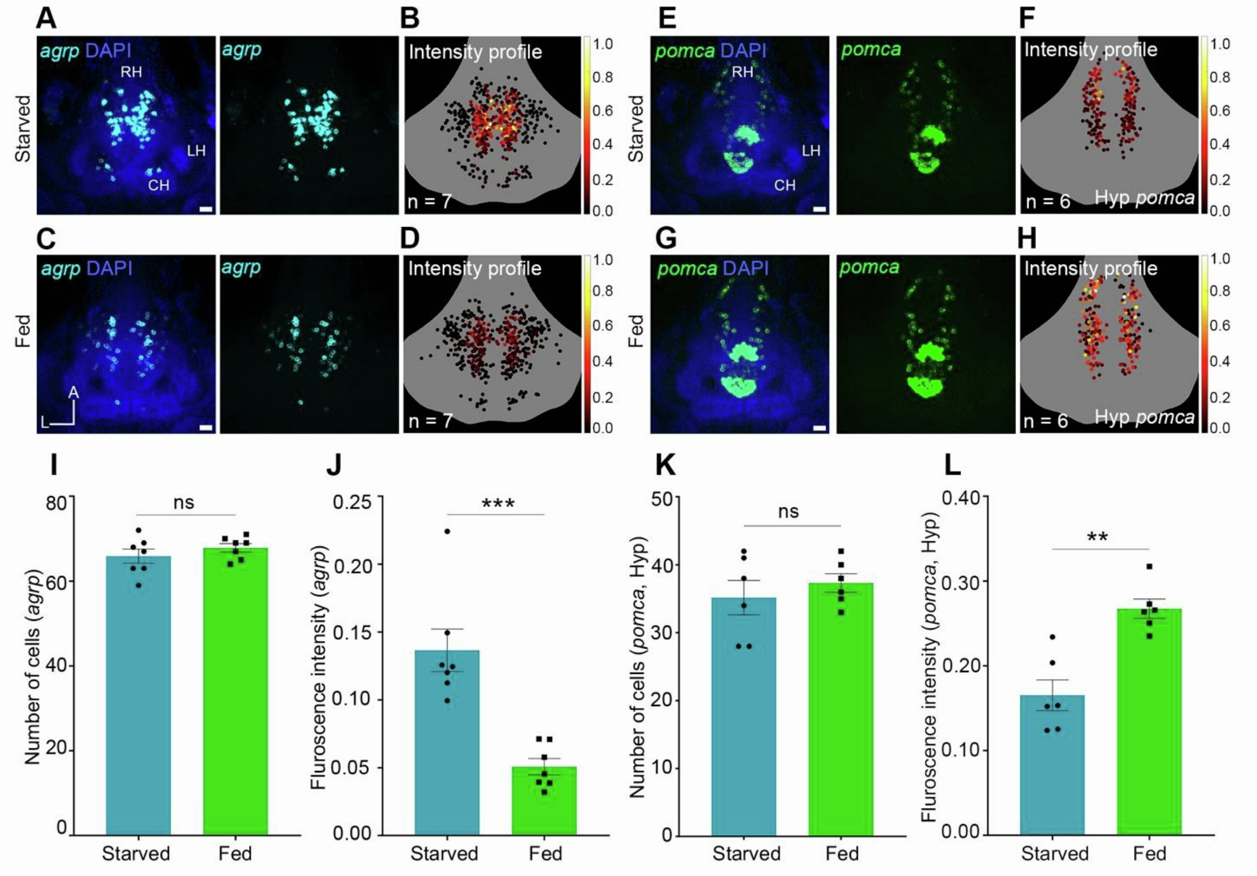

Figure S4. Changes in the expression of *pomca* and *agrp* in fed and starved conditions. Related to figure 2.

(A-D) *agrp* expression in the hypothalamus of starved (A-B) and fed (C-D) 7 dpf larvae. Normalized fluorescence intensity profiles for n=7 larvae are shown in B and D, respectively, with color bar indicated on the right. Positions of neurons were overlaid on a reference image (grey region) obtained from DAPI staining in larva used for the same experiment. RH: rostral hypothalamus, LH: lateral hypothalamus, CH: caudal hypothalamus. Scale bar, 20µm. (E-H) *pomca* expression in the hypothalamus and pituitary of starved (E-F) and fed (G-H) 7 dpf larvae. Normalized intensity profiles (only hypothalamic neurons) for n=6 larvae are shown in F and H, respectively, with color bar indicated on the right. Positions of neurons were overlaid on a reference image (grey region) obtained from DAPI staining in larva used for the same experiment.

(I-J) Graphs depicting number of cells expressing *agrp* (I) and the average normalized fluorescence intensity (J) in starved and fed larvae. n = 7 fish per group shown as individual data points.

(K-L) Graphs depicting number of hypothalamic cells expressing *pomca* (K) and the average normalized fluorescence intensity (L) in starved and fed larvae. n = 6 fish per group. Mann-Whitney's two-tailed t-test (I-L) *, $p < 0.05$, **, $p < 0.01$, ***, $p < 0.001$, ns, not significant. Dots represent individual larvae. Data are presented as mean ± SEM.

Figure S5. Related to Figure 2

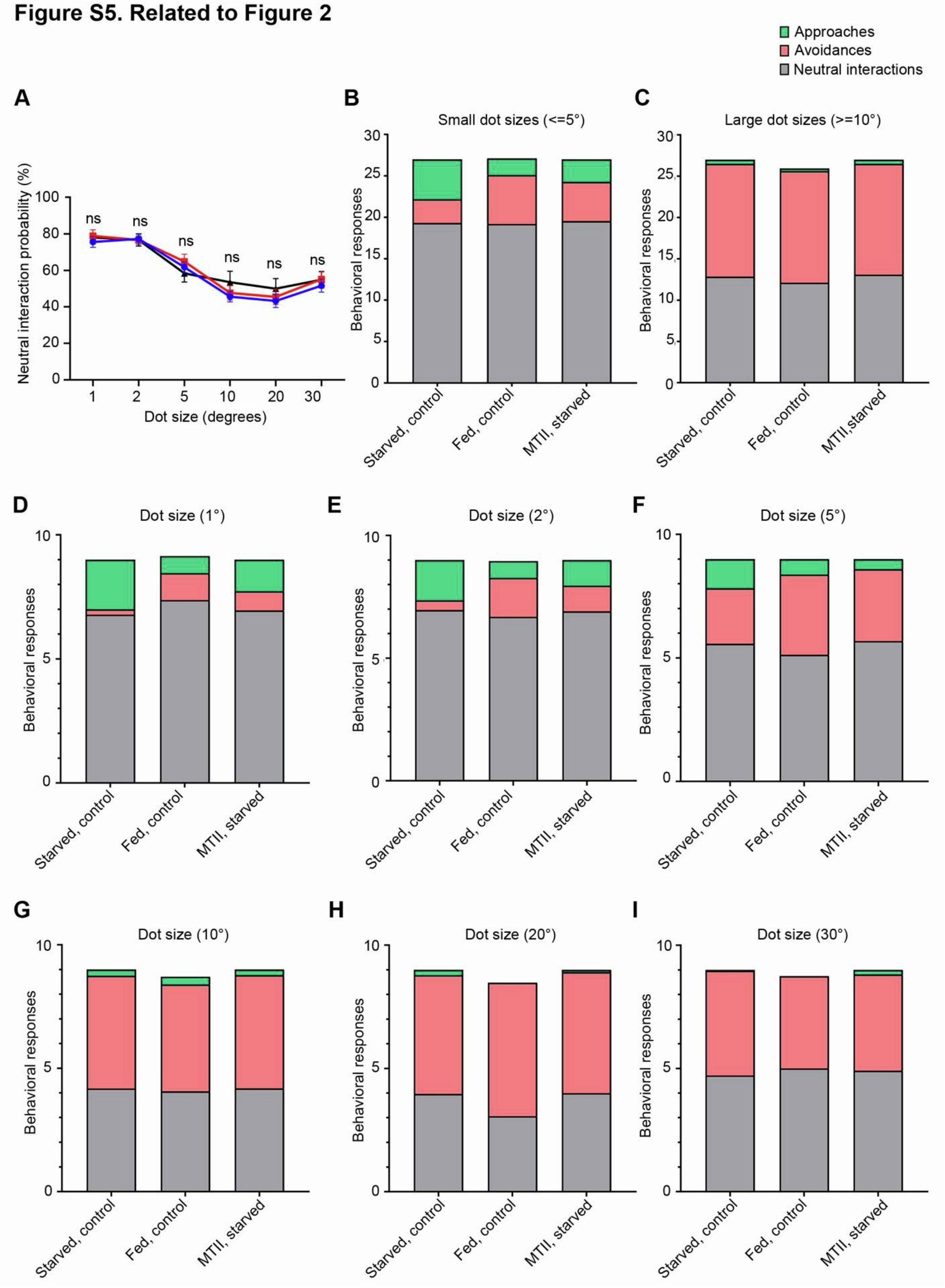

Figure S5. Behavioral Responses to Individual dot sizes. Related to figure 2.
(A) Graphs depicting neutral interaction probability in starved, fed, and starved MTII treated larvae. Data presented as mean±SEM. ns, not significant. T-test with Benjamini-Hochberg correction. For p values, see Table 1.
(B-I) Graphs depicting the average behavioral responses to small (1-5°), large (10-30°), and individual dot sizes (1°, 2°, 5°, 10°, 20° and 30°), respectively. $n_{starved,control}$ = 28, $n_{fed,control}$ = 16, $n_{starved,MTII\ treated}$ = 22.

Figure S6. Related to Figure 3

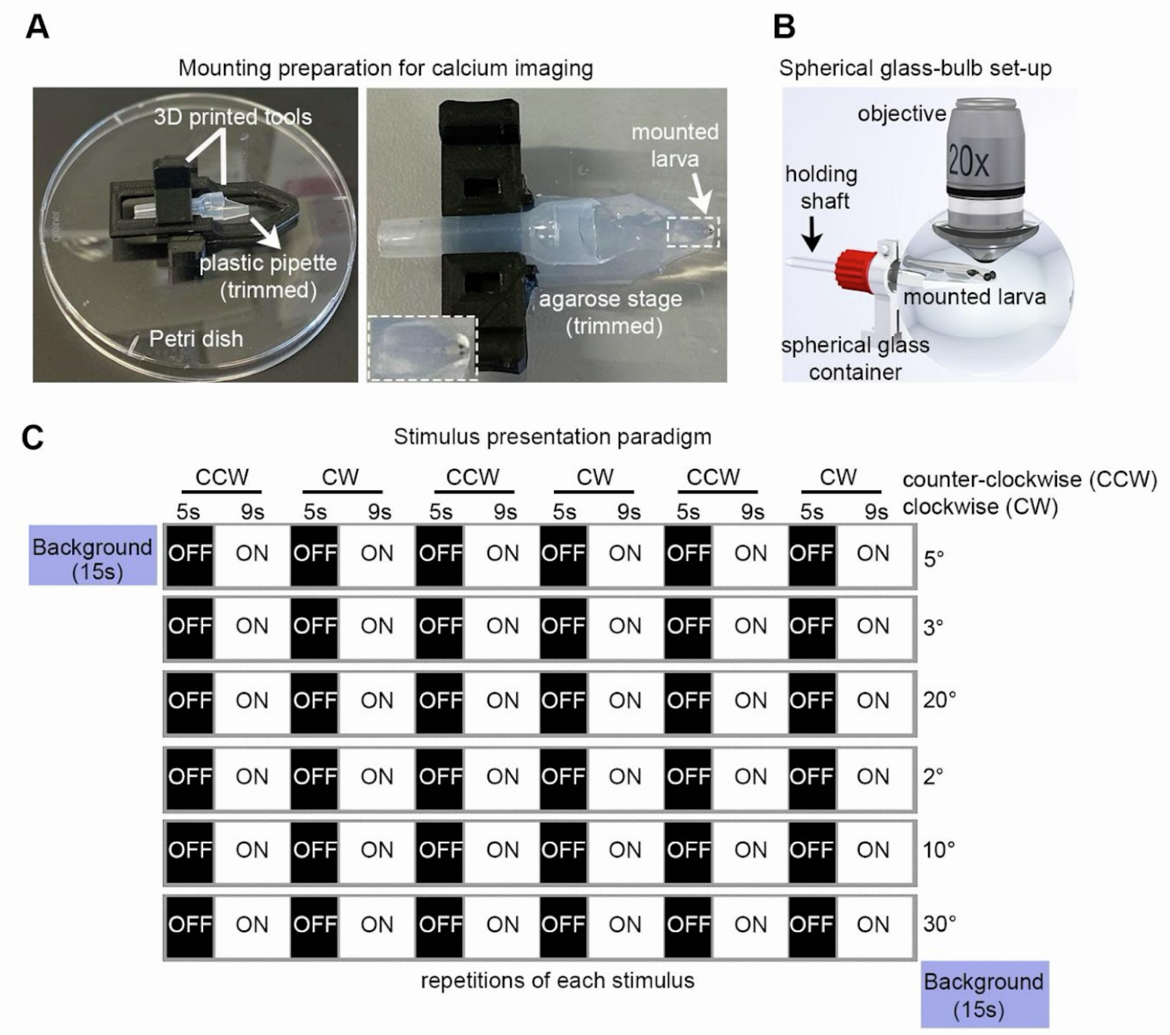

Figure S6. Mounting setup and stimulus paradigm used for calcium imaging. Related to figure 3.
(A) Images represent custom designed tools used for mounting larvae in low melting agarose.
(B) Schematic illustration of zebrafish larva placed in a glass-bulb setup used for calcium imaging. Note that the used spherical container was coated white so that it could serve as a stimulus screen (and was not transparent as depicted here).
(C) Stimulus presentation protocol. Bright circular moving dots (2°-30°) were presented on a dark background. Each 9 s stimulus (ON) phase was preceded by a 5 s pause (OFF) phase. During the pause phase, the stimulus was not in motion and only background was visible to the animal.

Figure S7. Related to Figure 3

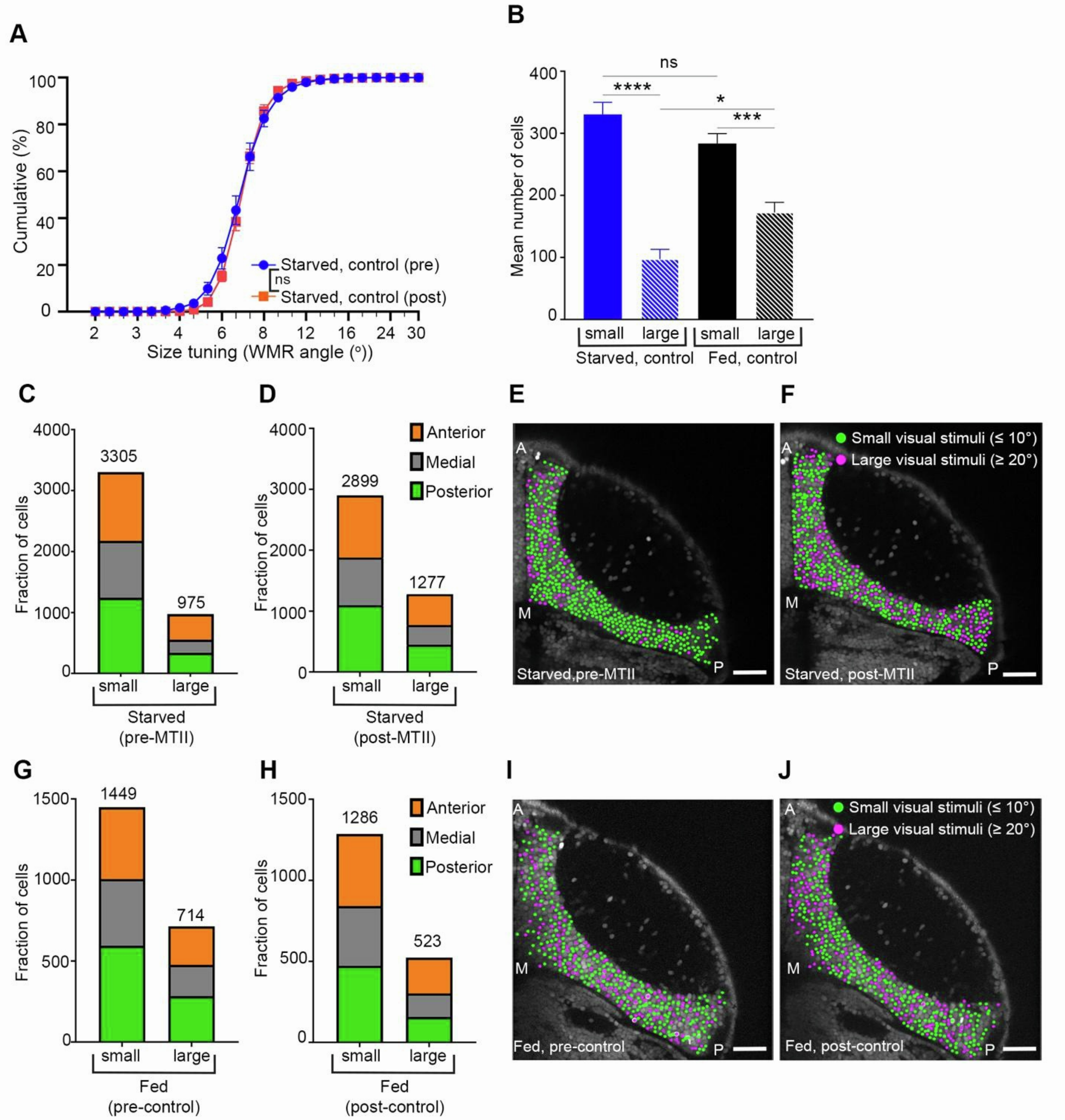

Figure S7. Calcium activity in untreated control larvae and shift in response of tectal PVNs to visual stimuli along A-P axis upon MTII treatment. Related to figure 3.

(A) Cumulative percentages of WMR angles of PVNs recorded before and after mock-treatment in starved (control) larvae at 7 dpf (n = 4 fish). In this experiment, starved larvae were not treated with the drug but were mounted in agarose and placed in embryo medium for the same duration as the drug treatment.

(B) Bar graphs representing number of cells responding to small (≤10°) and large (≥20°) visual stimuli in the tectum under starved (B) and fed conditions (C) respectively. n = 7, starved fish; n = 5, fed fish.

(C-D) Bar graphs representing number of cells tuned to small (≤ 10°) and large (≥ 20°) visual stimuli segregated along A-P axis of the tectum before and after MTII-treatment of starved larvae, respectively. n = 7 fish.

(E-F) Images representing position of small vs. large dot-responsive cells of an individual fish and overlaid on the H2B-GCaMP fluorescence (grey).

(G-H) Bar graphs representing number of cells tuned to small (≤ 10°) and large (≥ 20°) visual stimuli segregated along A-P axis of the tectum in fed larvae before and after mock-treatment, respectively. n = 3 fish.

(I-J) Images representing position of small vs. large dot-responsive cells of an individual fish and overlaid on the H2B-GCaMP fluorescence (grey). Scale bar, 50 µm.

*, $p < 0.05$, ***, $p < 0.001$, ****, $p < 0.0001$; ns, not significant; Paired Wilcoxon signed-rank test was performed on median WMR angle (defined as the WMR angle at which 50% neurons were responsive) derived from the cumulative WMR distributions (A), two-way ANOVA, followed by Tukey's multiple-comparisons test (B). Data are presented as mean ± SEM (A-B).

Figure S8. Related to Figure 3

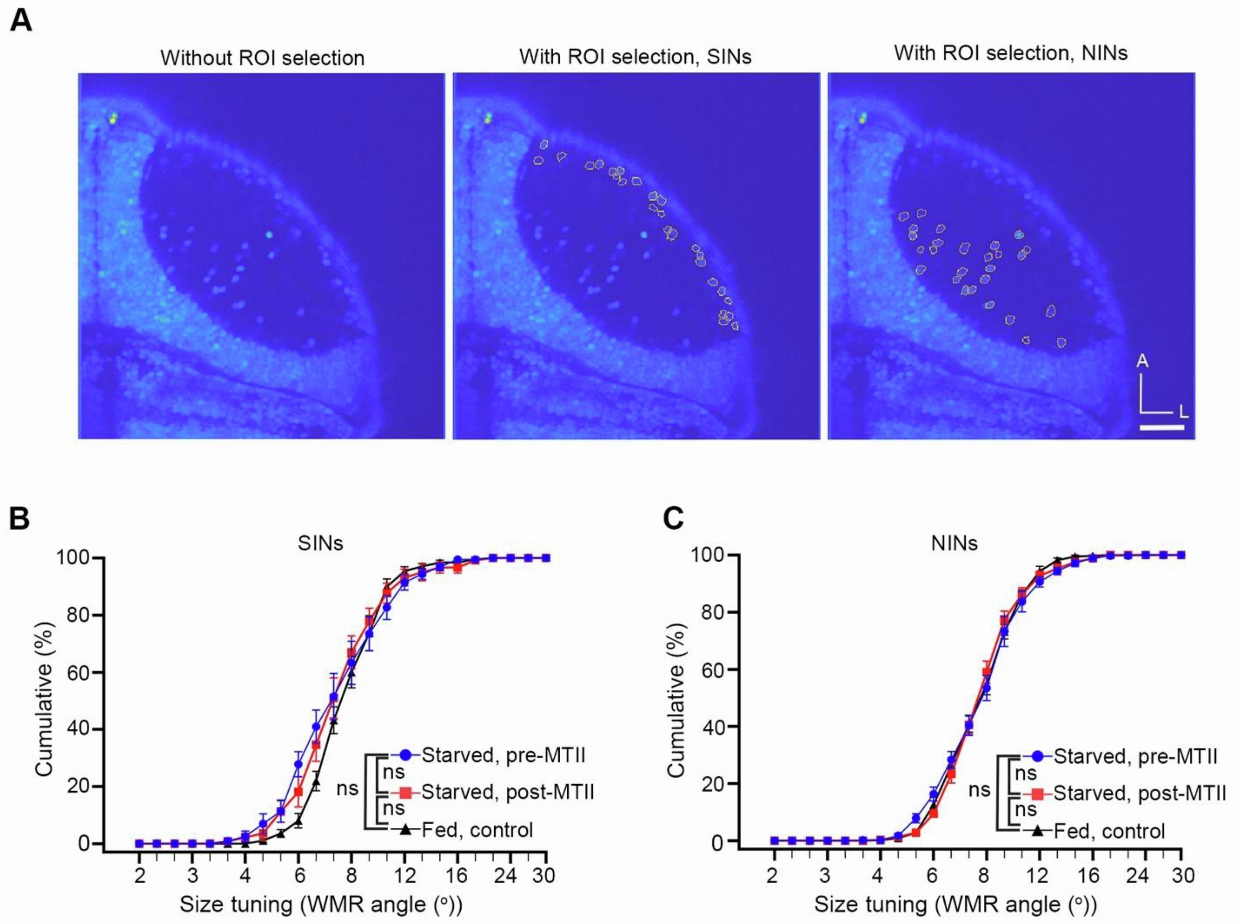

Figure S8: Calcium activity in superficial and neuropil interneurons (SINs and NINs). Related to figure 3.

(A) Images showing SINs (middle) or NINs (right) selected for analysis of calcium imaging in *elavl3*:*H2B-GCaMP6f* at 7 dpf. Scale bar, 50 µm.

(B-C) Cumulative percentages of WMR angles of SINs (B) and NINs (C) in *elavl3:H2B-GCaMP6f* larvae before and after MTII treatment in starved (n = 7 fish) and in control fed (n = 5 fish) conditions.

ns, not significant; Paired Wilcoxon signed-rank test was performed on median WMR angle (defined as the WMR at which 50% neurons were responsive) derived from the cumulative WMR distributions or Mann-Whitney's test was used for independent groups. Data are presented as mean ± SEM (B-C).

Figure S9. Related to Figure 4

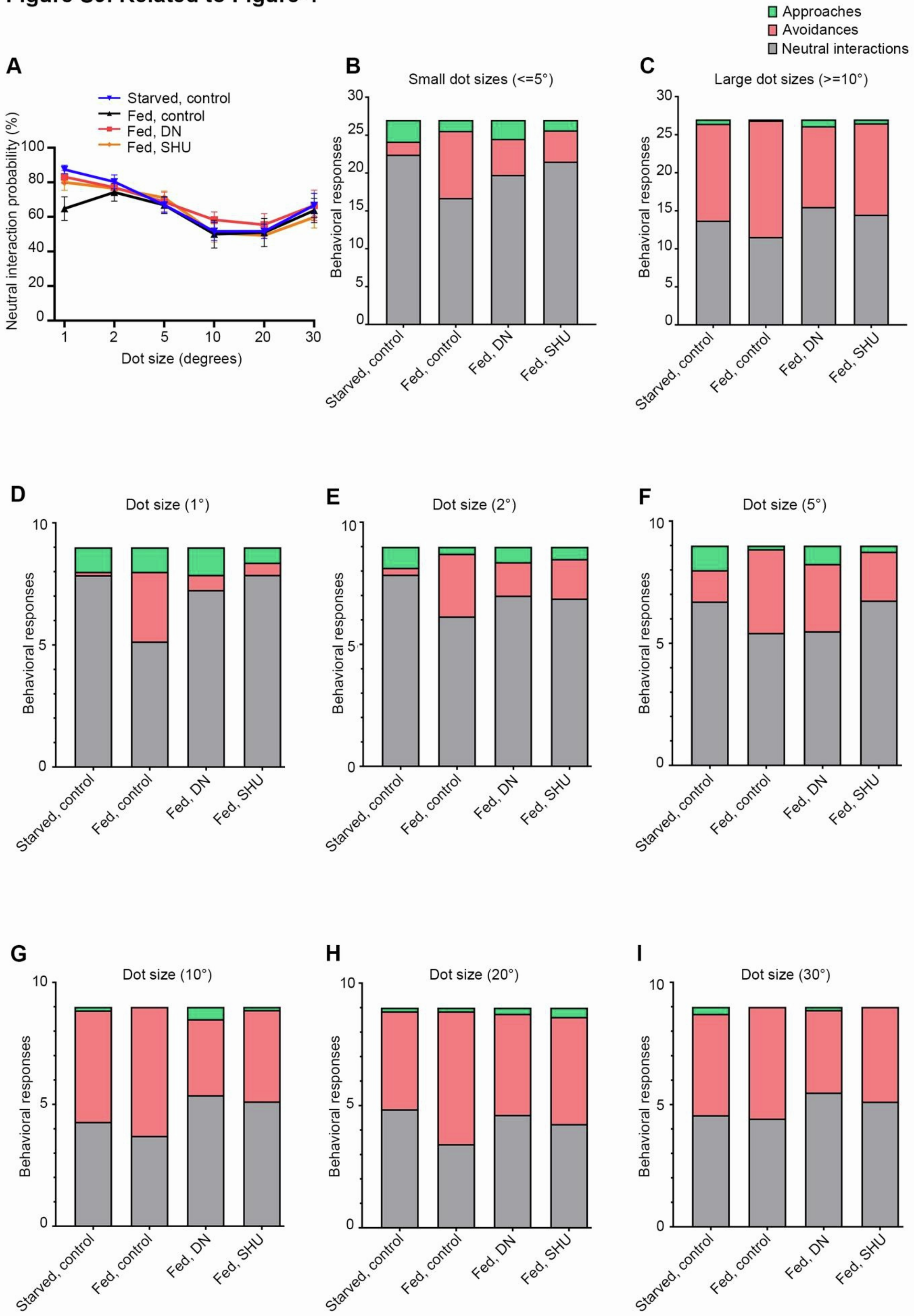

Figure S9. Behavioral responses to individual dot sizes. Related to figure 4.
(A) Dependence of neutral interaction probability on dot size in 7 dpf larvae for the following conditions and genotypes: starved and fed *s1013t:Gal4 UAS:GCaMP6s*, fed *s1013t:Gal4 UAS:GCaMP6s UAS:DNmc4r*, fed *s1013t:Gal4 UAS:GCaMP6s* treated with SHU9119. For p values, see Table 2.
(B-I) Graphs depicting the average behavioral responses to small (1-5°), large (10-30°), and individual dot sizes (1°, 2°, 5°, 10°, 20° and 30°), respectively. Data presented as mean±SEM. n = 12 to 16 larvae per group.

Figure S10. Related to Figure 4

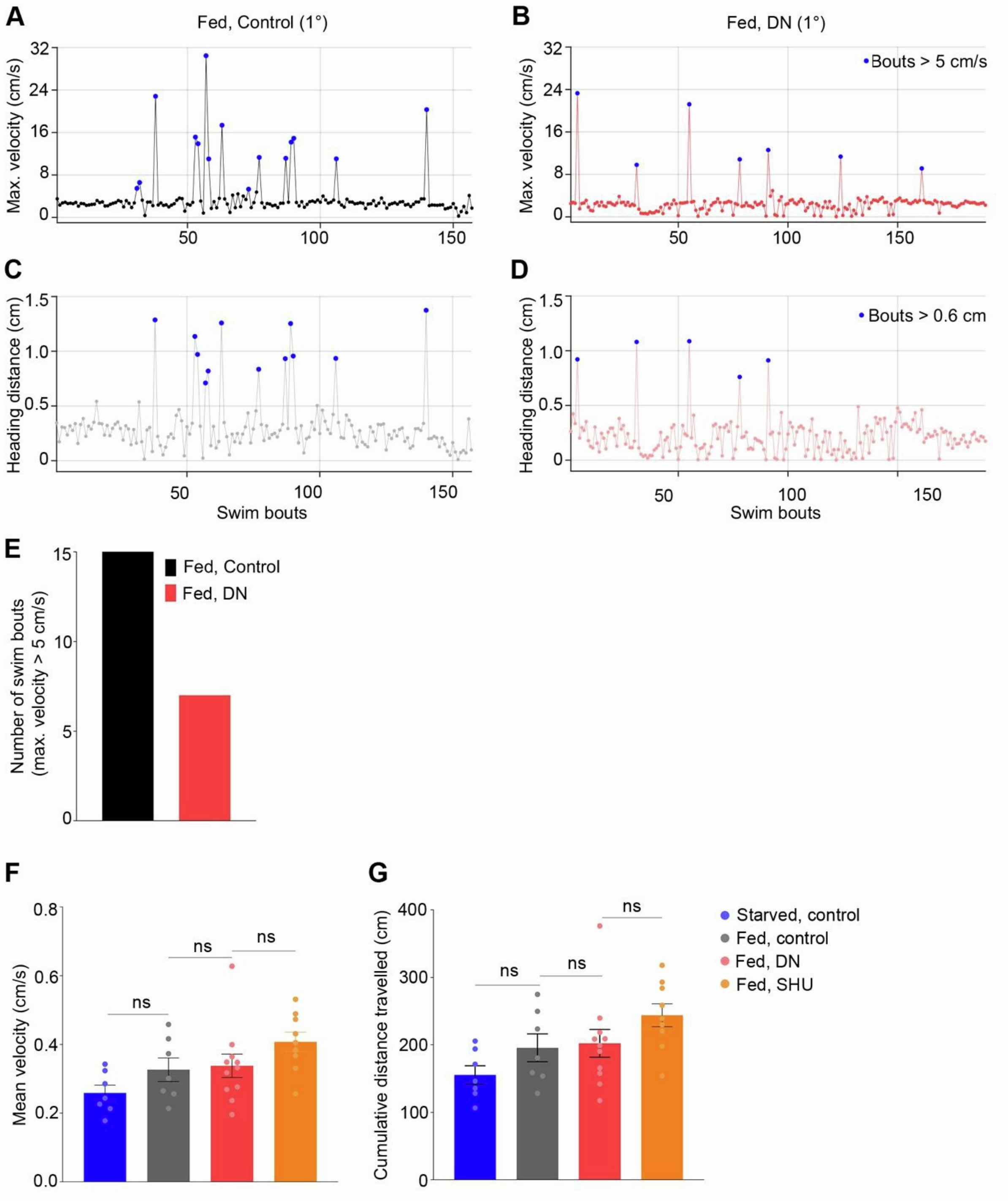

Figure S10. Automated behavior analysis for loss-of-function experiments. Related to figure 4.
(A) Maximum velocity for each swim bout in fed control larvae presented with small (1°) stimuli during size-discrimination assay. Blue dots indicate bouts exceeding the velocity threshold (>5cm/s). Each dot indicates a swim bout. n = 11 fish.

(B) Maximum velocity for each swim bout in fed dominant negative (DN) larvae presented with small (1°) stimuli during size-discrimination assay. Blue dots indicate bouts exceeding the velocity threshold (>5 cm/s). Each dot indicates a swim bout. n = 11 fish.

C) Heading distance calculated as the distance covered by the fish during a swim bout in fed control larvae presented with small (1°) stimuli during size-discrimination assay. Blue dots indicate bouts exceeding the threshold (> 0.6 cm). Each dot indicates a swim bout. n = 11 fish.

D) Heading distance calculated as the distance covered by the fish during a swim bout in fed DN larvae presented with small (1°) stimuli during size-discrimination assay. Blue dots indicate bouts exceeding the threshold (> 0.6 cm). Each dot indicates a swim bout. n = 11 fish.

E) Number of swim bouts with maximum velocity >5cm/s in fed control and fed DN fish, quantified from data points exceeding threshold (blue dots) shown in (A) and (B).

F) Mean swimming velocity (cm/s) during a 10 min recording. Bars represent mean ± SEM. Individual dots indicate single animals. Statistical comparisons using Mann-Whitney's test, ns, not significant. $n_{controlstarved} = 7$, $n_{controlfed} = 7$, $n_{s1013t:Gal4:UAS:\ DNmc4rfed} = 11$, $n_{SHU9119fed} = 9$.

G) Cumulative distance traveled (cm) during a 10 min recording. Bars represent mean ± SEM. Individual dots indicate single animals. Statistical comparisons using Mann-Whitney's test, ns, not significant. $n_{controlstarved} = 7$, $n_{controlfed} = 7$, $n_{s1013t:Gal4:UAS:\ DNmc4rfed} = 11$, $n_{SHU9119fed} = 9$.
